# Feasibility and Acceptability of a Mobile Health Exercise Intervention for Inactive Adults: 3-Arm Randomized Controlled Pilot Trial

**DOI:** 10.2196/52428

**Published:** 2024-08-09

**Authors:** Jacqueline Kiwata Dawson, Alison Ede, Madeleine Phan, Alec Sequeira, Hsiang-Ling Teng, Ayla Donlin

**Affiliations:** 1 Department of Physical Therapy California State University, Long Beach Long Beach, CA United States; 2 Department of Kinesiology California State University, Long Beach Long Beach, CA United States; 3 Department of Chemistry and Biochemistry California State University, Long Beach Long Beach, CA United States; 4 LifeFit Center Department of Kinesiology California State University, Long Beach Long Beach, CA United States

**Keywords:** digital health, physical activity, user experience, heart rate monitor, group exercise, mHealth, wearable, group exercise, feasibility, acceptability, mobile health, mobile health exercise, exercise, adults, randomized controlled trial, exercise program, support, wearables, screening, effectiveness, videoconference

## Abstract

**Background:**

Objective monitoring of self-directed physical activity (PA) is a common approach used in both fitness and health settings to promote exercise behavior, but adherence has been poor. Newer mobile health (mHealth) technologies could be a cost-effective approach to broadening accessibility and providing support for PA behavior change; yet, the optimal method of delivery of such interventions is still unclear.

**Objective:**

This study aimed to determine the feasibility and acceptability of an mHealth exercise intervention delivered in combination with objective monitoring in 3 ways: health education emails, asynchronous exercise videos, or synchronous videoconference exercise classes.

**Methods:**

Physically inactive (<30 min/wk) adults (cisgender women aged 31.5, SD 11.3 years, cisgender men aged 34.1, SD 28.9 years, and nonbinary individuals aged 22.0, SD 0 years) were randomized (1:1:1) to 8 weeks of increasing PA behavioral support: level 1 (health education+objective monitoring, n=26), level 2 (asynchronous contact, level 1+prerecorded exercise videos, n=30), or level 3 (synchronous contact, level 1+videoconference group exercise, n=28). Participants used a heart rate monitor during exercise and a mobile app for interaction. Primary outcomes were feasibility (accrual, retention, and adherence) and acceptability (user experience survey). Secondary outcomes assessed at baseline and 8 weeks included resting heart rate, self-reported PA, and quality of life. The exercise dose was evaluated throughout the intervention.

**Results:**

Between August 2020 and August 2021, 204 adults were screened for eligibility. Out of 135 eligible participants, 84 (62%) enrolled in the study. Retention was 50% (13/26) in level 1, 60% (18/30) in level 2 and 82% (23/28) in level 3, while adherence was 31% (8/26) in level 1, 40% (12/30) in level 2 and 75% (21/28) in level 3. A total of 83% (70/84) of the study sample completed the intervention, but low response rates (64%, 54/84) were observed postintervention at week-8 assessments. Program satisfaction was highest in participants receiving exercise videos (level 2, 80%, 8/10) or exercise classes (level 3, 80%, 12/15), while only 63% (5/8) of level 1 reported the program as enjoyable. Level 3 was most likely to recommend the program (87%, 13/15), compared to 80% (8/10) in level 2 and 46% (5/8) in level 1. Self-reported PA significantly increased from baseline to intervention in level 3 (*P*<.001) and level 2 (*P*=.003), with no change in level 1. Level 3 appeared to exercise at higher doses throughout the intervention.

**Conclusions:**

Only the videoconference exercise class intervention met feasibility criteria, although postintervention response rates were low across all groups. Both videoconference and prerecorded videos had good acceptability, while objective monitoring and health education alone were not feasible or acceptable. Future studies are needed to examine the effectiveness of videoconference exercise interventions on health-related outcomes during nonpandemic times and how asynchronous interventions might maximize adherence.

**Trial Registration:**

ClinicalTrials.gov NCT05192421; https://clinicaltrials.gov/study/NCT05192421

## Introduction

Regular physical activity (PA) is key to the prevention and management of noncommunicable diseases such as cardiometabolic, cancer, and chronic respiratory illnesses [1]. Yet, 28% of the worldwide adult population and 43% of adults in Western countries fail to meet current recommendations of 150 minutes of moderate or 75 minutes of vigorous intensity PA per week [2]. Reasons for poor PA adherence have centered around lack of resources, time, social support, and motivation, although the relative importance of each barrier has been shown to vary by socioeconomic status [3].

Digital health exercise programs are a promising strategy for addressing barriers to exercise by broadening accessibility, improving time efficiency, and increasing motivation [4]. Mobile health (mHealth) is a subset of digital health that includes technologies such as wearable sensors, smart devices, and mobile apps. Previous investigations have used these technologies to increase awareness of PA behavior through objective monitoring and to enhance the delivery of self-directed or remotely supervised exercise through asynchronous or synchronous implementations [5-7]. Objective monitoring devices such as pedometers, accelerometers, or heart rate (HR) monitors are purported to motivate participants to perform PA through the tracking of exercise-related behaviors [5,6]. With asynchronous interventions, participants are typically provided with instructor-led, prerecorded exercise videos that are delivered over web- or app-based platforms such as YouTube. Asynchronous interventions also use email or text reminders to push participants to perform certain behaviors, although participants decide when and where to engage in PA [6,7]. In contrast, synchronous exercise delivery requires participants to join a videoconference platform such as Zoom (Zoom Video Communications) at the same time as health and fitness professionals and other peers. In this way, participants can engage with peers and interact with health and fitness professionals in real time, facilitating a more personalized or community-like approach [7]. Thus, each technology confers specific advantages in persuading exercise behavior, such that technologies are often used together and in combination with health promotion education.

Previous mHealth exercise studies have predominantly used objective monitoring to promote exercise-related behaviors, including PA participation and adherence, overall PA time, weekly moderate-to-vigorous intensity physical activity (MVPA) time, walking time, and HR-based exercise dose [5,8,9]. Social Cognitive Theory is the theoretical framework that has commonly underpinned these interventions, as the theory focuses on how individuals can construct their environment to facilitate engagement of PA behavior [10]. Indeed, a recent systematic review of mHealth exercise interventions [8] identified multiple social cognitive theory constructs used in these interventions, including self-regulation through activity tracking and goal-setting for PA behavior. However, this review and others [5,8,9] did not find strong evidence of objective monitoring increasing PA, even when combined with health education materials, as more than minimal contact is likely needed to improve long-term PA adherence. Hence, additional behavioral support through asynchronous components, such as self-directed, home-based video use through websites or mobile apps, has been combined with objective monitoring. While these asynchronous interventions are slightly more effective at increasing adherence and eliciting PA behavior change [8,9], the greatest increases in PA have been observed when interventions include a synchronous component such as text messaging or a phone call with study personnel [8,9]. As posited by social cognitive theory, these synchronous components not only provide an informational benefit but also have the potential to improve motivation through social support. Social support and social persuasion, in the form of feedback and encouragement, along with vicarious experiences comprised of modeling by professionals, are influential sources of self-efficacy and subsequently motivation and behavior change [11]. As there is ample support that efficacy beliefs can be targeted to improve exercise behavior [12-14], combining efficacy beliefs with strategies to improve self-regulation (eg, learning to plan, set goals, and monitor one’s own workouts) is purported to enhance PA promotion [15].

The additional support from a synchronous component is also impactful for clinical outcomes, as improvements in glycemic control in individuals with type 2 diabetes [7], weight management in obese individuals [16], and exercise capacity across numerous chronic diseases [17] have been reported in systematic reviews. Of the synchronous modalities, videoconferencing has shown the greatest efficacy in improving PA and clinical outcomes, whether in the form of remote monthly check-ins or weekly exercise sessions with a health professional [7,16,17]. Video conferencing provides participants with the ability to learn through observing an instructor and possibly others taking part in an exercise session. However, less research has been conducted on videoconferencing as a modality to deliver remote group exercise sessions, despite its popularity during the COVID-19 pandemic and potential to initiate and adhere to PA [18].

A handful of investigations have used videoconferencing to deliver resistance, aerobic, or multimodal exercise in a group format using a randomized controlled design [19-25]. Of these investigations, only 3 [20,21,25] have assessed the feasibility of the intervention beyond attendance rates and evaluated the acceptability of the intervention qualitatively. Furthermore, only one of these interventions has been directed at sedentary but apparently healthy men and women [20], as Mascarenhas et al [21] focused on mothers and Hickman et al [25] on liver transplant recipients. Given the high rates of participant dropout, poor adherence, or missing outcome data reported in previous synchronous mHealth investigations [19,23,24], more information on the feasibility and acceptability of mHealth interventions with videoconferencing is needed. Further, reporting compliance with the prescribed intensities and durations of the intervention would enhance understanding of intervention effects, yet reporting exercise exposure is lacking in all but one study [20].

Therefore, the primary aim of this study was to determine the feasibility and acceptability of an 8-week mHealth progressive exercise intervention for physically inactive adults, designed as a pilot randomized controlled trial (RCT) with 3 levels of behavioral support. A secondary aim explored changes in resting HR, compliance through exercise dose, self-reported PA, and quality of life (QOL).

## Methods

### Study Design and Recruitment

We conducted a 3-arm randomized pilot trial of an 8-week intervention for physically inactive adults (ClinicalTrials.gov NCT05192421) that randomized participants (1:1:1) to increasing levels of behavioral support (level 1—minimal contact: lifestyle education+objective monitoring, level 2—asynchronous exercise videos+lifestyle education+objective monitoring, level 3—synchronous videoconference exercise classes+lifestyle education+objective monitoring).

Recruitment occurred between August 2020 and August 2021, with data collection ending in December 2021. Physically inactive adults (<30 min of MVPA per week) aged ≥18 years were recruited through Facebook and Instagram social media posts. During eligibility screening, levels of PA were verified using the International Physical Activity Questionnaire (IPAQ) [26], while potential contraindications to exercise were assessed using the American College of Sports Medicine exercise preparticipation questionnaire [27]. Eligible participants were required to have a mobile device with high-speed internet access and the ability to understand English. Individuals with a history of unstable cardiac or pulmonary disease, current use of tricyclic antidepressant or clozapine medications, or orthopedic issues that impaired walking were excluded from participation in the study.

### Ethical Considerations

All study procedures were approved by the California State University, Long Beach institutional review board (protocol number 1542178-7). All participants provided written consent prior to randomization, and all study data were deidentified prior to analysis. Participants were provided a $25 gift card as compensation upon completion of all study measurements.

### Randomization and Blinding

Participants were allocated (1:1:1) to increasing levels of behavioral support (levels 1-3) using computer-generated block randomization with blocks that randomly varied in sizes of 3, 6, 9, or 12. The randomization list was prepared in advance by an investigator (HLT) uninvolved with any other study procedures. After consent and enrollment procedures, the allocation assignment was requested from HLT. Participants were told that they would be randomly assigned to receive certain resources but were unaware of what was received relative to other study arms.

### Intervention

Social cognitive theory [10] and group dynamics [28] provided the conceptual frameworks for the 8-week intervention. Specifically, the constructs of social support, self-efficacy, self-regulation, and observational learning were incorporated throughout the intervention to facilitate PA behavior (Table 1) [11-15,28,29].

**Table 1:** Mapping of theoretical constructs to components of a 3-arm, 8-week technology-supported physical activity intervention for physically inactive adults.

Theoretical construct and intervention component		Level 1	Level 2	Level 3
**Social support [28]**				
	Interaction with other study participants through the social feed on the MyZone app	✓	✓	✓
	Weekly progress emails from study team	✓	✓	✓
	Interaction with study coach and peers during classes			✓
**Self-efficacy [11-15]**				
	Social modeling and persuasion from participating with study group on the MyZone app	✓	✓	✓
	Feedback through progress emails on meeting weekly PA^a^ recommendations	✓	✓	✓
	Feedback and encouragement from instructors during classes			✓
**Self-regulation [15]**				
	Information about exercise sessions (effort, progress) through MyZone heart rate monitor and app	✓	✓	✓
	Instruction from professional		✓	✓
**Observational learning [29]**				
	Health education emails and website posts	✓	✓	✓
	Exercise videos		✓	
	Exercise classes			✓

^a^PA: physical activity.

Study arms had increasing layers of interpersonal contact [30], with level 1 representing the lowest level of contact and level 3 receiving the most behavioral support. Specifically, level 1 received a control level of educational content on diet and exercise consisting of information that can be found publicly on the United States Department of Health and Human Services Office of Disease Prevention and Health Promotion website [31]. The static educational information was adapted for use on the study website (Multimedia Appendix 1) and also delivered in a weekly email to participants (Multimedia Appendix 2). Each week covered a different topic on diet and exercise education but did not contain specific exercise advice or exercise progressions. Level 2 was able to access the same static website content as level 1, and received the same weekly educational email as level 1, along with 3 prerecorded videos every week of recommended exercise, accessible directly in the email or through the study website. Level 3 received the same resources provided to level 1, along with 3 instructor-led group exercise classes per week that were attended by 5-8 level 3 participants over a cloud-based video conference (Zoom).

All participants were provided a HR monitoring device (MyZone MZ-3) to track exercise intensity. The MZ-3 is a commercially available wearable fitness technology that has increased in use in recent years to stream live effort data during exercise sessions, with the purported benefit of increased social interaction and accountability [32-34]. The MZ-3 estimates maximal HR (MHR) using the equation 211–(0.64×age) [35] and categorizes minute-by-minute HR into one of 5 zones based on %MHR, similar to other popular devices such as the Polar HR monitor [36]. Each zone corresponds to a score [37] known as a MyZone effort point (MEP=%HRmax×duration), such that zone 1: 50%-59% MHR, 1 MEP/minute; zone 2: 60%-69% MHR, 2 MEP/minute; zone 3: 70%-79% MHR, 3 MEP/minute; zone 4: 80%-89% MHR, 4 MEP/minute; zone 5: 90%-100% MHR, 4 MEP/minute. Moderate-intensity PA (64%-76% MHR) consists of exercising in zones 2 and 3, while vigorous-intensity PA (77%-93% MHR) corresponds to zones 3-5 [27]. Accumulating 300 MEPs/week meets national PA guidelines of 150 minutes/week of moderate-to-vigorous activity or 75 minutes/week of ≥vigorous activity [31]. When wearing the HR monitor, participants can view real-time HR data through the MyZone mobile app, which is also uploaded automatically to the user’s cloud account. Following each session, the app summarizes the total MEPs earned per workout on a social feed that is viewable only by other participants in the same study arm. The study team accessed the HR data weekly to download the data and calculate the MEPs earned per week.

#### Study Website and Weekly Emails

All participants received the same weekly email that summarized their activity in MEPs from the previous week, reminded them to engage in at least 3 sessions/week of PA toward the recommended 300 MEPs while wearing the HR monitor, encouraged interaction with other participants in their group via the Myzone app, and provided educational content on a lifestyle-related topic. The email was intended to assist participants in developing self-regulatory skills, as seeing their past successes from week to week could allow for the development of positive past performance, the most important source of self-efficacy. Educational topics and an example email can be found in Multimedia Appendix 2. In addition to the workout summary and educational content, emails contained links to the study website that were tailored to the participant’s group assignment. Specifically, level 2 received links to exercise videos that were posted on the website, while level 3 received a link to the next videoconference exercise class. Level 1 emails did not contain additional links. When accessing the website, participants used a username and password that were provided following orientation to the MZ-3 and MyZone apps. The study website contained all resources referenced in the weekly email and was tailored to each participant’s group assignment (Multimedia Appendix 1).

#### High-Intensity Functional Training Sessions

Level 2 (asynchronous exercise videos) and level 3 (synchronous videoconference exercise class) participants received a 35-minute high-intensity functional training (HIFT) session 3 times/week for 8 weeks. HIFT refers to a multimodal style of training that incorporates both aerobic and muscle-strengthening exercises through functional, multijoint movements [38]. Although referred to as “high intensity,” HIFT can be modified to any fitness level. In this intervention, intensity began at a moderate intensity (70% MHR) in accordance with American College of Sports Medicine guidelines for apparently healthy adults [27] and gradually progressed in a structured fashion to vigorous intensity (85% MHR) for safety and intervention reproducibility (Table 2). A duration of 8 weeks was selected to match a similar HIFT investigation targeting cardiometabolic risk factors in overweight and obese individuals [39]. Each session was led by an instructor and included a 5-minute warm-up, a 25-minute conditioning, and a 5-minute cool-down focused on core and flexibility exercises. The dynamic warmup and cool-down were repeated every session, while the conditioning segment progressed in a linearly periodized manner. Exercises were selected to emphasize functional movements [38] and progressed in complexity and format according to the periodization model. Each exercise session consisted of 9 body weight exercises for the dynamic warmup, 7 body weight or dumbbell exercises for the conditioning segment, 3 body weight exercises for core training, and 3 stretches for the cool-down (Multimedia Appendix 3). Participants were asked to monitor their intensity during the exercise sessions by wearing the HR monitor and using the Myzone app, with a target intensity during the conditioning component encouraged by the instructor.

**Table 2:** Linear periodization of the exercise program used in the videoconference exercise class (level 3) and asynchronous video (level 2) groups in an 8-week technology-supported intervention for physically inactive adults (macrocycle: 8 weeks).

Exercise focus	Multijoint acclimation	Multijoint, combination	Multijoint, multiplanar	Multijoint, ladder
Encouraged intensity (% maximum heart rate)	≥70%	≥75%	≥80%	≥85%
Mesocycle	Wk 1-2	Wk 3-4	Wk 5-6	Wk 7-8

### Primary Outcome: Feasibility and Acceptability

The primary outcome of the study was feasibility, which was assessed through accrual, retention and adherence, and acceptability, which was assessed through qualitative evaluation, as defined in previous guidelines [40]. Accrual was defined as the time between the enrollment of the first and last participant. Retention was defined as the percentage of participants completing postintervention assessments. Adherence was defined as the observed to prescribed number of total exercise sessions completed using the MZ-3 (3 sessions/wk×8 weeks=24 prescribed sessions). A priori, we considered the intervention feasible if the following criteria were satisfied [40]: (1) accrual, ≥50% of eligible participants enrolled into the trial in a 1-year period; (2) retention, ≥70% of enrolled participants completing postintervention assessments; and (3) adherence and completion of ≥70% prescribed sessions (≥16 sessions). The acceptability of the intervention was assessed through feedback obtained at the end of the 8-week intervention through an investigator-developed web-based user experience survey. The survey asked participants to evaluate perceived ease of use and usefulness of the mobile app, wearable sensor, emails, videos (level 2), classes (level 3), and overall satisfaction with the intervention through Likert scales (1=strongly agree, 5=strongly disagree) and open-ended questions (Multimedia Appendix 4).

### Secondary Outcomes

The intervention was developed to support PA behavior changes that have been associated with improvements in cardiometabolic risk factors [39]. Therefore, we explored changes in resting HR, self-reported PA level, QOL, and compliance as measured through exercise dose. All measurements were assessed at baseline and postintervention except for exercise dose, which was monitored continuously throughout the intervention.

#### Resting Heart Rate

Upon waking and after voiding the bladder, participants recorded a single 10-minute HR session in supine using the MZ-3 HR monitor and MyZone mobile app. The recording was uploaded to the participant’s cloud account and accessed by the study team.

#### Physical Activity

The IPAQ was used to quantify the duration, frequency, and intensity of leisure, occupational, commuting, and household activities through a 7-day recall [26]. The IPAQ has been shown to have acceptable measurement properties, including repeatable data (pooled *r*=0.81 across data from 12 countries) and criterion validity comparable to other self-report methods (pooled *r*=0.3). The IPAQ was administered remotely using a web-based survey application (Qualtrics).

#### Quality of Life

QOL was assessed through the Short Form-36 (SF-36) [41] and administered remotely using a web-based survey application (Qualtrics). The SF-36 consists of 4 physical and 4 mental health components that quantify QOL in 8 domains. Raw scores were transformed into a scale of 0-100 (0=worst, 100=best) consistent with the scoring instructions.

#### Exercise Dose and Compliance

The training dose of each exercise session was quantified from the MZ-3 HR monitor and MyZone mobile app using MEPs (intensity-minutes) where *Session MEPs=(target HR zone)×(min of exercise).* Total exercise dose for the intervention was calculated as the sum of session MEPs across the 8-week program, where *total exercise dose=Σ session MEPs*, while weekly exercise dose was calculated as the sum of session MEPs across 1 week of the intervention. Compliance was assessed by comparing the observed weekly exercise dose to the prescribed 300 weekly MEPs and the observed total exercise dose to the prescribed total MEPs across the intervention (300 MEPs×8 weeks=2400 MEPs total).

### Statistical Analysis

Because the data for most variables were not normally distributed, outcomes are presented as median (IQR) and nonparametric analyses were performed with a significance level set to ⍺=.05 (2-sided) except as indicated with Bonferroni correction for multiple comparisons. Descriptive statistics were used to summarize participant characteristics, feasibility, acceptability, and secondary outcomes. Proportions were calculated for categorical variables and median (IQR) for continuous variables. Comparisons of baseline characteristics and differences between groups were made using Kruskal-Wallis rank sum tests for continuous outcomes and Dunn tests with Bonferroni adjustment for post hoc testing. For within-group baseline to postintervention comparisons, intent-to-treat models were used to compute the difference (post-pre) on individual scores, and Wilcoxon signed rank tests with Bonferroni correction were used to assess significance. Analyses were performed in RStudio (version 1.2.1335; Posit Software).

### Power

The sample size for a feasibility pilot trial is recommended to be at least n=50 (25 participants per group) [42]. Our estimated sample size for this pilot study was 75 participants (25 participants per group). Accounting for 10% attrition, we recruited a sample size of 84.

## Results

### Participants

**Figure 1:**
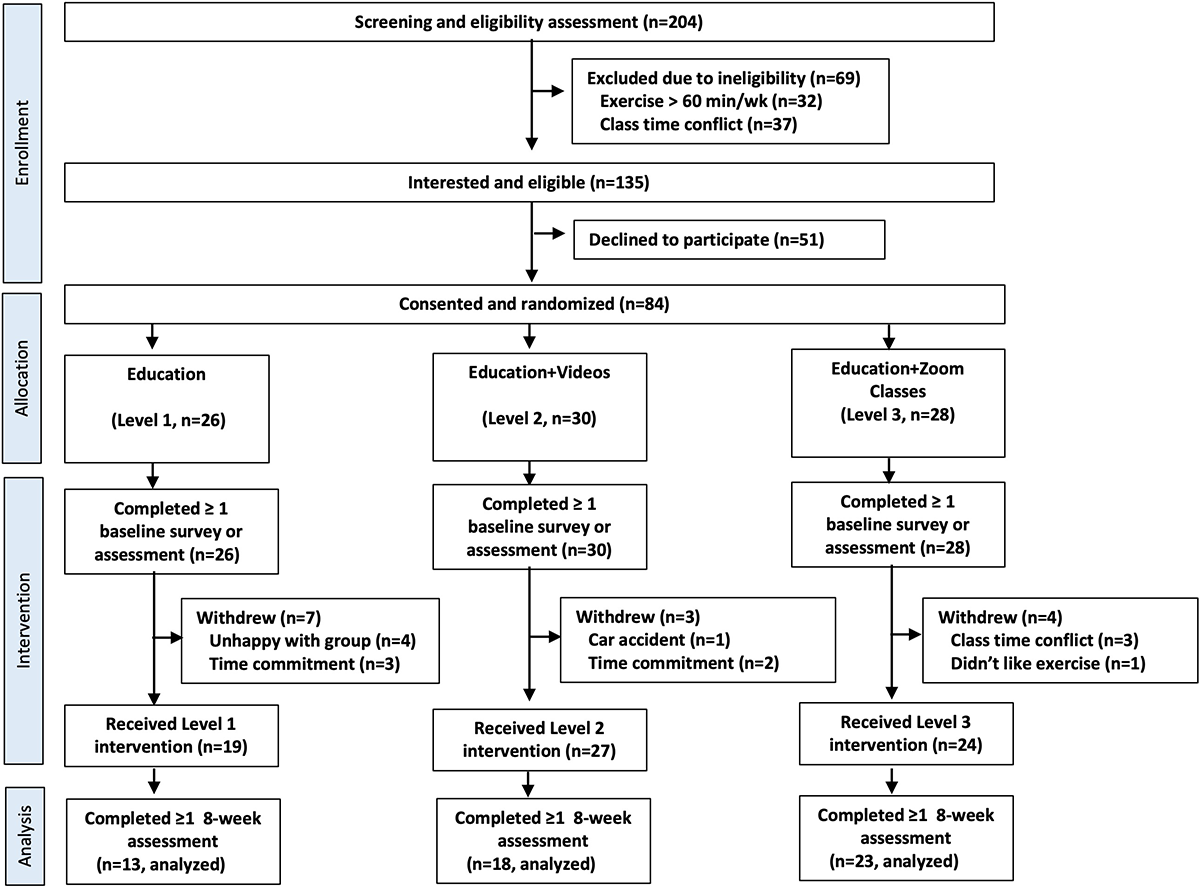
Consolidated Standards of Reporting Trials (CONSORT) diagram showing participant enrollment, allocation, and flow.

A total of 204 potential participants expressed interest in the study via a web form between August 2020 and August 2021 (Figure 1). Of these candidates, 135 were eligible and 84 were enrolled in the study. Out of the 84 consented participants, 26 (31%) were randomized to level 1, 30 (36%) to level 2, and 28 (33%) to level 3. A total of 70 (83%, 70/84) participants completed the 8-week intervention, which included wearing the MZ-3 fitness tracker up until the eighth week (n=19 in level 1, n=27 in level 2, and n=24 in level 3). For the 14 participants that withdrew after randomization, time commitment or schedule conflict were the primary reasons stated for withdrawal. Withdrawal across groups was n=7 in level 1, n=3 in level 2, and n=4 in level 3. Of the n=70 participants that completed the intervention, n=54 (64%, 54/84) completed at least 1 secondary outcome measure (eg, HR, IPAQ, and SF-36) at week 8.

Participants demographic and baseline characteristics are presented in Table 3. Of the 84 participants, the majority were cisgender women (77/84, 92%). No participants reported performing vigorous-intensity PA at baseline, while 31% (8/26) of level 1, 40% (12/30) of level 2, and 50% (14/28) of level 3 reported walking for PA for durations greater than 150 minutes/week (*P*=.87).

**Table 3:** Baseline self-reported demographic and physical characteristics of 84 physically inactive adults participating in an 8-week technology-supported physical activity intervention, overall, and by randomization level.

Characteristic		Level 1 (n=26)	Level 2 (n=30)	Level 3 (n=28)	Total (N=84)
Age (years), median (IQR)		27 (24-33)	30 (22-37)	31 (22-44)	29 (22-36)
**Gender**					
	Cisgender women, n (%)	24 (29)	27 (32)	26 (31)	77 (92)
	Cisgender men, n (%)	2 (2)	2 (4)	2 (2)	6 (8)
	Nonbinary, n (%)	0 (0)	1 (1)	0 (0)	1 (1)
BMI (kg/m^2^), median (IQR)		26.3 (23.1-29.7)	27.4 (24.3-32.3)	28.1 (24.1-33.1)	27.4 (24.1-31.2)

### Feasibility

We found accrual to be feasible, as 62% (84/135) of eligible participants enrolled in the study within a 1-year period. For retention, 50% (13/26) of level 1, 60% (18/30) of level 2, and 82% (23/28) of level 3 participants completed at least 1 postintervention assessment. As our threshold for retention was ≥70% of enrolled participants completing postintervention assessments, only the level 3 intervention met retention criteria. Last, we found adherence to be feasible only in level 3, as 75% (21/28) of level 3 participants completed at least 70% of the prescribed sessions (≥16 sessions). Adherence in levels 1 and 2 was low, with only 40% (12/30) of level 2 and 31% (8/26) of level 1 participants completing at least 16 exercise sessions over the 8-week intervention.

### Intervention Acceptability

**Figure 2:**
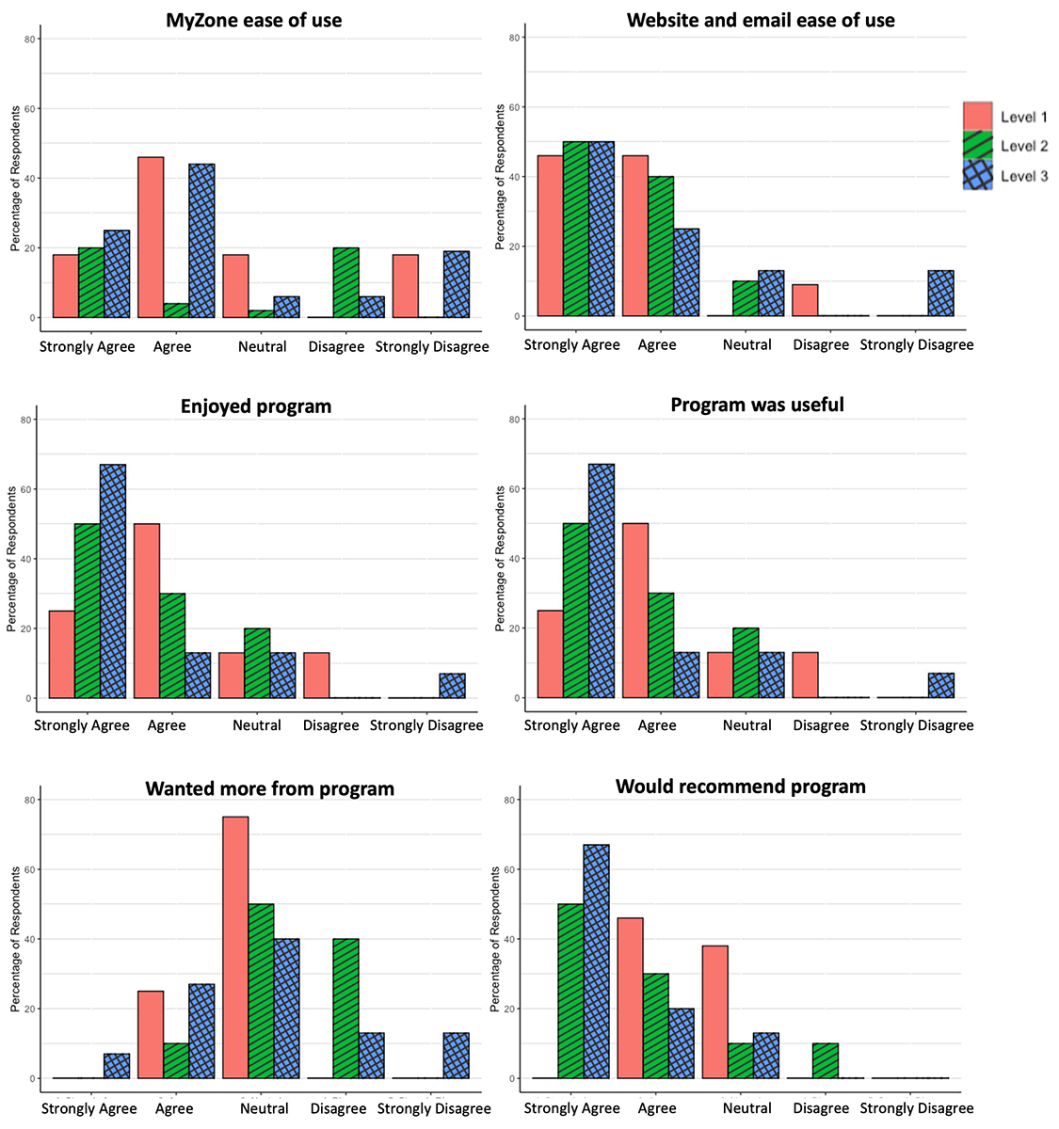
Acceptability of an 8-week remote exercise intervention in physically inactive adults participating in an 8-week technology-supported physical activity intervention, by randomization level.

Figure 2 presents results from the user experience survey, which was completed by 33 participants (n=8 level 1, n=10 level 2, n=15 level 3). Program satisfaction was higher in participants receiving exercise videos (level 2) or Zoom classes (level 3), with 80% (8/10) of level 2 and 80% (12/15) of level 3 reporting the program as enjoyable and useful. In contrast, 63% (5/8) of level 1 participants reported the program as enjoyable or useful. The greatest proportion of participants that wanted more from the program were from level 3 (34%, 5/15) rather than level 2 (10%, 1/10) or level 1 (25%, 2/8). Level 3 was also most likely to recommend the program to others (87%, 13/15), while 80% (8/10) of level 2 and only 46% (5/8) of level 1 indicated they would recommend the program.

Of the 33 participants who completed the user experience survey, 30 (n=6 level 1, n=10 level 2, and n=14 level 3) provided feedback on the program through open-ended questions. The qualitative data suggested that participants had different experiences depending on the intervention they received. The majority of level 3 participants (57%, 8/14) indicated what they liked most about the program was the exercise instruction from the coach during the live digital classes.

I liked the coaches because they were good motivators during the workouts. Even though the workouts were tough, I liked trying to keep up

Level 3

I enjoyed the instructors. Very encouraging and warm

Level 3

For level 2, most participants (50%, 5/10) indicated they liked the convenience of the video workouts.

I liked the videos a lot it gave me the flexibility to do it on my own time

Level 2

I liked that with the videos I could complete the workouts whenever convenient for me

Level 2

For level 1, most participants (50%, 3/6) indicated that the social support from the group was the element they enjoyed the most about the program.

I liked how I was able to interact with other within my study group

Level 1

Seeing the community work out made me feel competitive and wanted to work out more

Level 1

For participants who received the workouts (n=24, level 2 and level 3 pooled responses), 46% (10/24) commented that the format, variation, or exercises themselves were the most enjoyable aspects of the program.

I liked that the exercise videos followed a pattern of instruction so I wasn't confused each week. It was easy to follow along. It also was short so I felt like it was easier to accomplish

Level 2

The workouts targeted my whole body, so I feel the strength and change all throughout

Level 2

The program was well organized, easily accessible in a virtual format and quite motivating. Wonderful study!

Level 3

Regardless of group allocation, the most liked program element was the increased awareness of being physically active and the accountability provided by the intervention.

I generally feel more motivated to live a healthier lifestyle

Level 2

I really enjoyed the classes. It helped me to get back to being more active after COVID

Level 3

I liked being part of a group and that it made me think about my activities

Level 1

When asked what was disliked about the program, the most common response from any group was “no dislikes” (20%, 6/30, levels 1-3 pooled responses). Other common dislikes were time constraints or schedule conflicts that interfered with working out (17%, 5/30) and the format of some of the workouts (17%, 5/30).

I did not like working at my own pace for some of the workouts, I prefer having a number of reps to try and hit for every set of exercises. I find it difficult to keep myself motivated when everyone in the group is going at their own pace

Level 3

I did not like how when we had a ladder style program, I couldn't see the full list of exercises the second time around, so I couldn't push myself further because I was trying to remember what exercise to do next rather than just doing the next exercise in the cycle

Level 2

Most participants (73%, 22/30, levels 1-3 pooled responses) found the MyZone belt easy to use, but some indicated technical difficulties.

I had a lot of difficulty getting the myzone belt to work, but I liked the idea of using it and I liked the challenges and being part of a group

Level 1

I had trouble with using the belt as far as getting it to work, ie, finding the heart rate. Sometimes it would stop working in the middle of a workout and it wouldn't record my heart rate for a period of time that I worried affected my score

Level 2

The Myzone belts were nice but at times it wouldn't read the activity I was doing. Like in the middle of the workout it would stop

Level 3

The most common response when asked about recommendations for the program was “no changes” (43%, 13/30, levels 1-3 pooled responses). Other recommendations included more flexibility in the scheduling of classes (2/30), more variety in the exercises (2/30), more methods of engaging with group members (2/30), using a device without a chest strap (2/30), letting participants select their own music (1/30), and better camera quality for the videos (1/30).

### Self-Reported Physical Activity, Resting Heart Rate, and Quality of Life

Baseline, week 8 and within-group changes for self-reported PA, resting HR, and QOL are presented in Table 4 as median (IQR). For self-reported PA, weekly MVPA time increased from baseline to 8 weeks in level 2 (*P*=.003) and level 3 (*P*<.001), but not in level 1. No significant within-group changes were observed from baseline to week 8 for walking time, total PA time, resting HR, or QOL.

**Table 4:** Baseline, 8 weeks, and change from baseline for self-reported physical activity scores, resting heart rate, and quality of life in physically inactive adults participating in an 8-week technology-supported physical activity intervention, by randomization level.

		Level 1		Level 2		Level 3	
		N^a^	Median (IQR)	N^a^	Median (IQR)	N^a^	Median (IQR)
**Walking time^b^ (min/wk)**							
	Baseline	22	54.5 (16.3-275.0)	27	86.0 (17.5-195.0)	27	150.0 (30.0-432.5)
	8 Weeks	11	120.0 (55.0-377.5)	14	142.5 (88.6-275.0)	22	107.5 (40.0-315.0)
	Change, baseline to 8 weeks	11	45.0 (2.5-150.0)	14	45.0 (0-84.8)	22	–45.0 (–141.3 to 30.0)
**MVPA^c,d^ time (min/wk)**							
	Baseline	22	0.0 (0.0-36.4)	29	0.0 (0.0-0.0)	27	0.0 (0.0-0.0)
	8 Weeks	11	225.0 (115.0-480.0)	14	233.1 (76.9-406.7)	22	368.8 (218.1-624.7)
	Change, baseline to 8 weeks	11	147.5 (45.3-425.0)	14	156.3 (63.5-421.3)	22	347.5 (158.4-602.2)
**Total PA^e,f^ time (min/wk)**							
	Baseline	22	195.6 (80.9-532.5)	29	210.0 (93.6-652.5)	27	395.0 (151.9-755.0)
	8 Weeks	11	465.0 (265.0-861.3)	14	365.5 (181.9-622.8)	22	478.8 (283.8-845.5)
	Change, baseline to 8 wk	11	117.5 (–264.7 to 299.4)	14	112.5 (–34.4 to 291.3)	22	83.1 (–198.8-415.9)
**Resting HR^g,h^ (beats/min)**							
	Baseline	20	63.0 (62.0-69.3)	26	69.0 (59.5-81.5)	24	67.0 (60.0-76.0)
	8 Weeks	8	70.0 (63.0-77.5)	6	77.5 (67.0-83.5)	5	71.0 (53.0-74.0)
	Change, baseline to 8 wk	8	–2.5 (–10.5 to 11.8)	6	10.0 (–1.3 to 15.3)	5	–4.0 (–4.0 to –3.0)
**SF-36^i,j^**							
	Baseline	23	90.0 (77.5-95.0)	26	100.0 (91.3-100.0)	25	90.0 (80.0-95.0)
	8 Weeks	12	100.0 (91.3-100.0)	15	100.0 (87.5-100.0)	23	95.0 (95.0-100.0)
	Change, baseline to 8 weeks	12	2.5 (0.0-12.5)	15	0.0 (–7.5 to 0.0)	23	5.0 (0.0-10.0)
	Within-group (*P*)		.206		.482		.021

^a^Analyses were conducted on participants who completed measurements at baseline and 8 weeks. Not all participants exposed to the intervention completed each measurement.

^b^Level 1: *P*=.18; level 2: *P*=.14; and Level 3: *P*=.17.

^c^MVPA: moderate-to-vigorous intensity physical activity.

^d^Level 1: *P*=.03; level 2: *P*=.003 (Wilcoxon signed rank test with Bonferroni correction for multiple comparisons [*P*=.02]); and level 3: *P*<.001 (Wilcoxon signed rank test with Bonferroni correction for multiple comparisons [*P*=.02]).

^e^PA: physical activity.

^f^Level 1: *P*=.29; level 2: *P*=.20; and level 3: *P*=.11.

^g^HR: heart rate.

^h^Level 1: *P*=.94; level 2: *P*=.46; and level 3: *P*=.22.

^i^SF-36: Short Form-36.

^j^Level 1: *P*=.21; level 2: *P*=.48; and level 3: *P*=.02.

### Exercise Dose and Compliance

The total and weekly exercise dose throughout the intervention as objectively measured from the MZ-3 fitness tracker is presented in Table 5. Level 3 was observed to have the highest levels of time spent in exercise, moderate-vigorous intensity exercise, and number of exercise sessions. Compliance was also highest in level 3, with 39% (11/28) of participants meeting the 300 weekly prescribed MEPs and 2400 MEPs across the 8-week intervention. A total of 33% (10/30) of level 2 participants were classified as meeting weekly and total MEPs, while only 12% (3/26) of level 1 participants were identified as compliant.

**Table 5:** Total and weekly exercise dose as measured by the Myzone MZ-3 fitness tracker in physically inactive adults participating in an 8-week technology-supported physical activity intervention, by randomization level.

		N^a^	Median (IQR)
**Total exercise dose (MEPs^b^)**			
	Level 1	19	256.5 (0-1210.5)
	Level 2	27	1339.5 (178.8-2473.8)
	Level 3	24	2017.5 (1174.3-2768.8)
**Weekly exercise dose (MEPs)**			
	Level 1	19	32.1 (0-151.3)
	Level 2	27	167.5 (22.4-334.2)
	Level 3	24	252.2 (146.8-346.1)
**Total time in moderate-vigorous intensity (min)**			
	Level 1	19	81.0 (0-569.0)
	Level 2	27	414.0 (57.3-854.0)
	Level 3	24	767.0 (361.0-1050.0)
**Weekly time in moderate-vigorous intensity (min/wk)**			
	Level 1	19	9.0 (0-63.2)
	Level 2	27	46.0 (6.4-94.9)
	Level 3	24	85.2 (40.1-116.7)
**Total exercise sessions (count)**			
	Level 1	19	4.5 (0-16.8)
	Level 2	27	11.0 (2.3-28.0)
	Level 3	24	24.0 (15.8-28.0)

^a^Analyses were conducted on participants who completed the 8-week intervention.

^b^MEP: MyZone effort point

## Discussion

### Principal Results

The primary aim of this pilot, randomized controlled trial was to evaluate the feasibility and acceptability of an mHealth intervention with additive levels of behavioral support. The secondary aim explored differences in resting HR, self-reported PA, QOL within each group from baseline to week 8, and the descriptively assessed exercise dose performed by each intervention level. In total, 83% (70/84) of the study sample completed the intervention, but we observed low response rates at the week 8 assessments, with only 64% (54/84) of the study sample completing acceptability or secondary outcomes. We found that the intervention level with the most support (level 3: videoconference exercise class+health education+HR monitoring) was the only feasible arm, as level 3 met retention (≥70% of enrolled participants completing postintervention assessments) and adherence (≥70% completion of the prescribed 16 sessions) criteria. Intervention acceptability was similar between level 3 and level 2 (exercise videos+health education+HR monitoring), with 80% of participants in both groups indicating program enjoyment, although only 53% (15/28) of level 3 and 33% (10/30) of level 2 participants completed acceptability assessments. Level 1 (health education+HR monitoring) had the lowest behavioral support, feasibility, acceptability, and week-8 response rate (31%, 8/26).

In addition to being classified as the most feasible and acceptable, the level 3 intervention had the highest compliance (39% vs 12% level 1 and 33% level 2), the greatest increase in self-reported MVPA from baseline to postintervention (median 348, IQR 158-602 min/wk vs median 148, IQR 45-425 min/wk level 1 vs median 157, IQR 64-421 min/wk level 2), and appeared to perform the most exercise throughout the intervention (median 2018, IQR 1167-2810 MEPs vs median 257, IQR 0-1258 MEPs level 1 vs median 1340, IQR 154-2558 MEPs level 2). The synchronous and supervised nature of level 3 likely underlies why this level was more successful than the other intervention levels. Videoconferencing provides the advantage of real-time interactions, which has the potential to improve motivation through social support and enhance self-efficacy. As posited by social cognitive theory, leveraging social support through interactions with other participants and enhancing self-efficacy through social modeling and feedback from the class instructor can positively influence PA behavior [11]. Exercise adherence and PA engagement are purported to be enhanced with group interactions [28] and social support from peers [43], which has been demonstrated in previous mHealth investigations targeting healthy older adults [22,44], where high adherence and satisfaction were reported in participants receiving group exercise classes via videoconference. Qualitative feedback from the acceptability results reinforces this assertion, as many level 3 participants indicated that support from the class instructor was important in facilitating motivation to exercise, as was the support from other study participants in the class. However, a primary concern stated by level 3 participants was the lack of flexibility and time conflict with the class scheduling. In addition, the level 3 intervention was more labor-intensive and required a fitness coach to be available 3 times per week for 8 weeks. These schedule- and resource-related concerns reinforce disadvantages cited in a previous review on synchronous interventions [45]. Therefore, there is a need to identify how group strategies can increase PA behaviors in videoconference-delivered exercise interventions while still allowing for flexibility in scheduling and resource allocation.

Although the level 2 intervention was not deemed feasible based on poor adherence rates, intervention acceptability was similar between level 3 and level 2, and level 2 participants significantly increased time spent in MVPA from baseline to week 8 (median 156, IQR 64, 421). Participants consistently cited the convenience and flexibility of performing the exercises on their own schedule as instrumental in engaging in PA, which agrees with findings from systematic reviews on asynchronous mHealth interventions [7,45]. Yet, the absence of direct, real-time supervision may explain the low adherence throughout the intervention, as newly active participants may require more feedback and guidance to advance their exercise training. This is supported by findings from a similar mHealth intervention [20], where lower adherence and compliance were observed in participants who received asynchronous exercise videos compared to those who also received health coaching videoconferencing. In addition, asynchronous designs may benefit from taking participant preferences into account, as feedback from level 2 participants indicated more versatility in music, exercise format, and program structure. Given that the unsupervised, asynchronous format is a popular format used in health care settings for preventative and outpatient rehabilitative programs, future studies that use asynchronous delivery should consider user-centered approaches such as community-based participatory research in developing the intervention.

We are not aware of other studies in inactive adults comparing the feasibility and acceptability of synchronous videoconference group exercise to asynchronous video-demonstrated exercise and unsupervised objective monitoring. Thus, a strength of our design was the inclusion of 3 mHealth strategies that are commonly used in health care and fitness settings. Participants who received more resources (level 3 and level 2) had higher adherence, lower attrition, and reported greater satisfaction with the intervention, which agrees with previous research using similar designs [20,30]. However, behavioral support, rather than mode of delivery, appears to be the more important determinant of adherence. In the present study, all 3 groups leveraged self-regulation through the fitness tracker and observational learning through the health education tips, but only the group that received multiple types of behavioral strategies (ie, social support, self-efficacy, self-regulation, and observational learning) increased PA engagement. Similar findings have been reported in previous mHealth interventions, where providing the same support across in person versus digital interventions demonstrated no difference in outcomes [19,22]. In addition, when some level of coaching or interaction is added to asynchronous exercise or remote monitoring, adherence improves [30], while objective monitoring with no human interaction is largely ineffective at inducing behavioral change [5]. Thus, our results suggest that some amount of human interaction is important to successfully modify exercise behavior in remotely delivered programs.

Only 1 investigation using videoconference with exercise [20] has also reported compliance, or the extent to which participants complied with the prescribed intensities and durations of exercise. Given that physiological adaptations have a dose-response relationship to exercise, quantifying the dose of exercise performed during an intervention can help define which exposures are effective for sustaining participation and evoking physiological changes [37]. We found compliance in performing exercise sessions to the prescribed intensity and durations to be 39% (11/28) in level 3, 33% (10/30) in level 2, and 12% (3/26) in level 1. In contrast, adherence, as calculated by the number of exercise sessions per week, was much higher, with 75% (21/28) in level 3, 40% (12/30) in level 2, and 31% (8/26) in level 1. These findings agree with Bannell et al [20], who reported a greater proportion of adherent participants than compliant participants in their mHealth intervention. While neither this trial nor the investigation from Bannell et al [20] aimed to evaluate the effectiveness of the intervention on health-related outcomes, the significance of exercise dose lies in its effect on physiologic response. Miller et al [37] found no difference in change in health-related outcomes between adherent and compliant individuals compared to non-adherent but compliant participants. This led the authors to conclude that cumulative exposure to exercise intensity and duration mediates physiological change rather than simple attendance at exercise sessions. Taken together, these findings underscore the importance of defining adherence beyond attendance, and that quantifying compliance in mHealth exercise interventions is warranted to better understand physiological adaptations.

### Limitations

There are several limitations to this study that may have influenced the findings. The first limitation of this trial was the low and uneven completion of postintervention assessments across all groups. This may have influenced acceptability findings, especially as only participants with positive experiences may have provided feedback. Second, the individuals who volunteered for the study were primarily young-to-middle aged adults with access to mobile devices and high-speed internet. Further, recruitment was conducted over Facebook and Instagram social media posts, which may have created a selection bias for participants with the literacy, access, and desire to use these technologies and participate in a remote intervention. Thus, the results may not be representative of all adults, especially those with low technology literacy or access. Next, because the study was conducted during the pandemic when in-person fitness experiences were limited or nonexistent, this could positively skew results in favor of remote interventions. As such, our results may not be generalizable to non-pandemic eras, and future work is needed to determine if videoconference interventions enhance PA behavior to the same extent as in-person experiences. Another limitation is that we did not include a second method of objectively measuring PA besides the HR monitor. Because PA adherence was only recorded when the HR monitor was worn, assessment of PA through a continuously worn accelerometer could have provided additional insight into non-exercise PA. Thus, the PA recorded in this study was likely an underestimate of overall activity. Another limitation is the lack of assessment of health-related outcomes. While the primary aim of this study was feasibility, measurement of clinical outcomes could provide effect sizes for a fully powered RCT. Last, a follow-up assessment after the intervention would have been helpful to evaluate the sustainability of PA adherence beyond the 8-week intervention.

### Conclusions

In summary, our randomized controlled pilot trial demonstrates that a videoconference mHealth intervention is feasible and can promote increases in PA. Future, appropriately powered trials should investigate the effectiveness of videoconference interventions on health-related outcomes in comparison to in-person interventions. More research is needed to elucidate feasible exercise interventions using asynchronous components, especially in diverse populations.

## Appendix

Multimedia Appendix 1 Study website and example resources for each level.

Multimedia Appendix 2 Weekly email topics and sample email.

Multimedia Appendix 3 Exercise intervention details.

Multimedia Appendix 4 User experience survey and response coding.

Multimedia Appendix 5 CONSORT-eHealth checklist (V 1.6.1).

## Abbreviations

HIFT: high-intensity functional training
HR: heart rate
IPAQ: International Physical Activity Questionnaire
IQR: interquartile range
MEP: Myzone effort point
mHealth: mobile health
MHR: maximal heart rate
MVPA: moderate-to-vigorous intensity physical activity
PA: physical activity
QOL: quality of life
RCT: randomized controlled trial
SF-36: Short Form-36

## Data Availability

The data sets generated during this study are available from the corresponding author on reasonable request.
