# Supplementary material for: Feasibility and Acceptability of a Mobile Health Exercise Intervention for Inactive Adults: 3-Arm Randomized Controlled Pilot Trial
Source: JMIR Form Res. 2024 Aug 9;8:e52428. doi: 10.2196/52428 (PMC11346126; doi:10.2196/52428)

**Multimedia Appendix 1. Study website**

1. Study Website Home Page – Public access


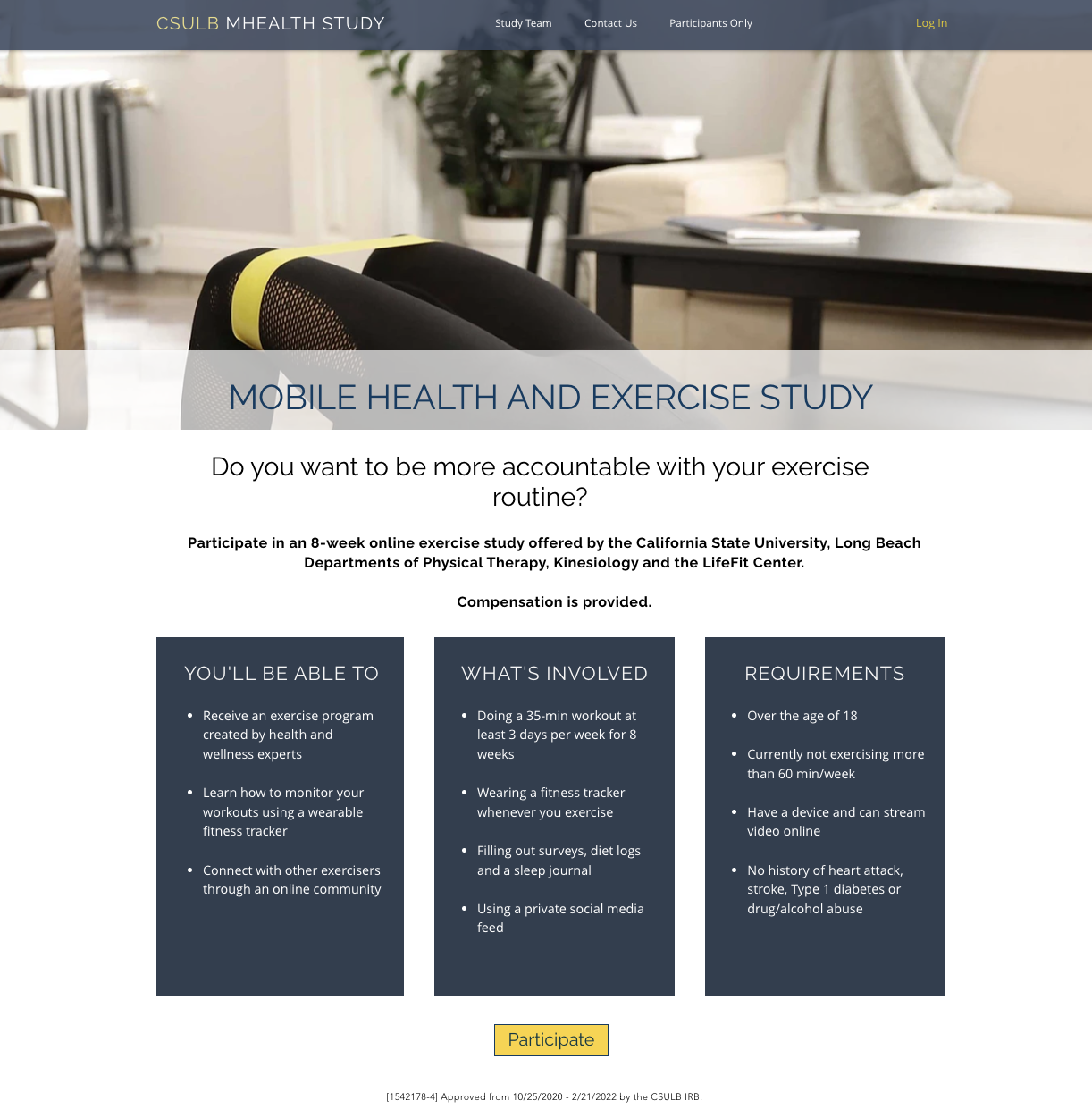


1. Study Website Log In – Accessed by Level 1, Level 2 and Level 3 participants


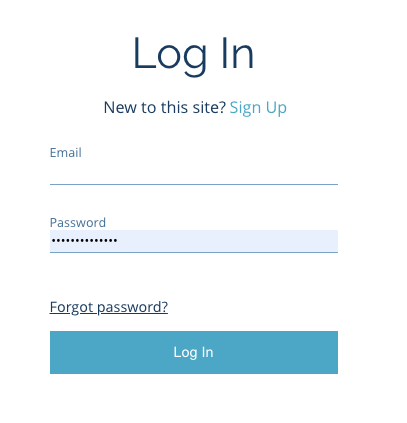


Study Website Onboarding - Accessed by Level 1, Level 2 and Level 3 participants


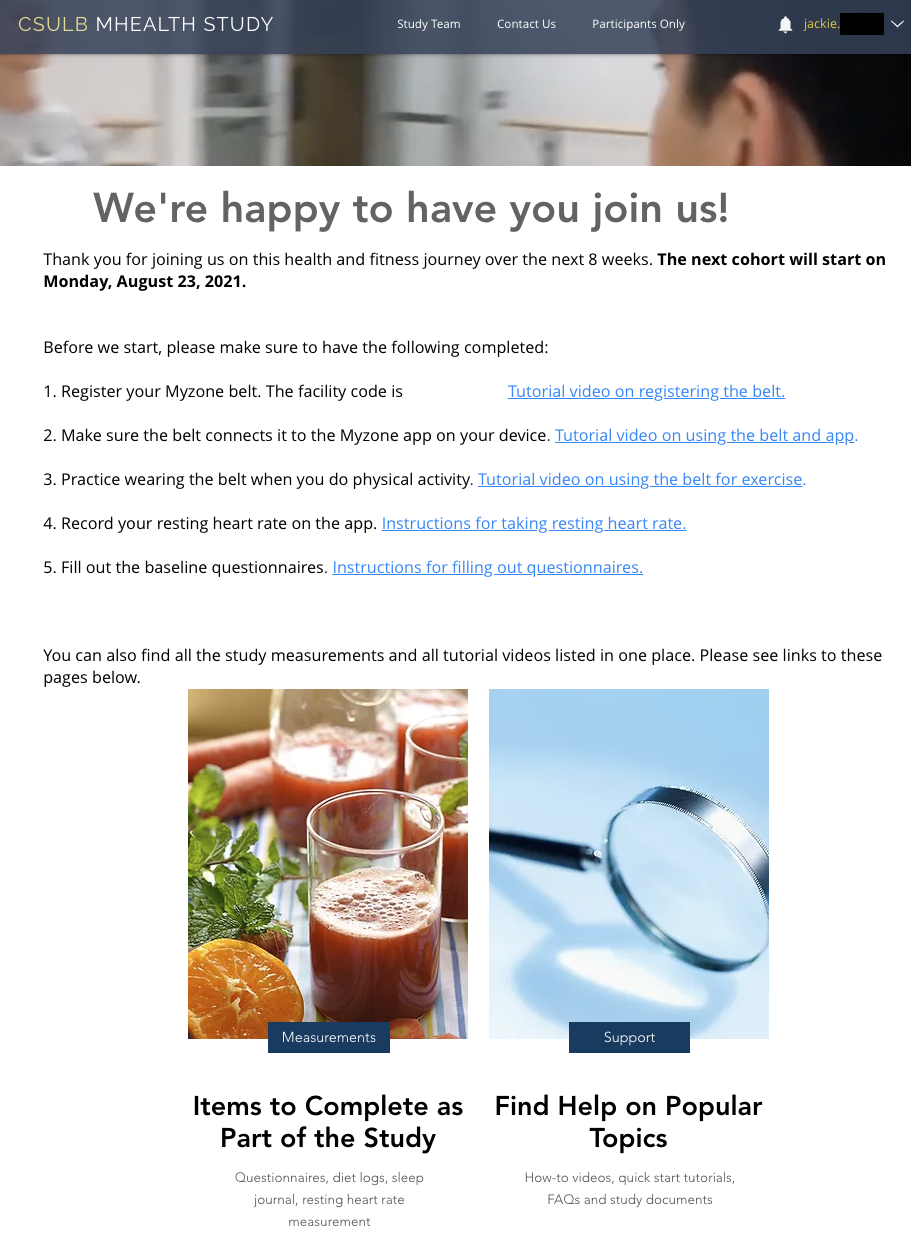


1. Study Measurements Explanation - Accessed by Level 1, Level 2 and Level 3 participants


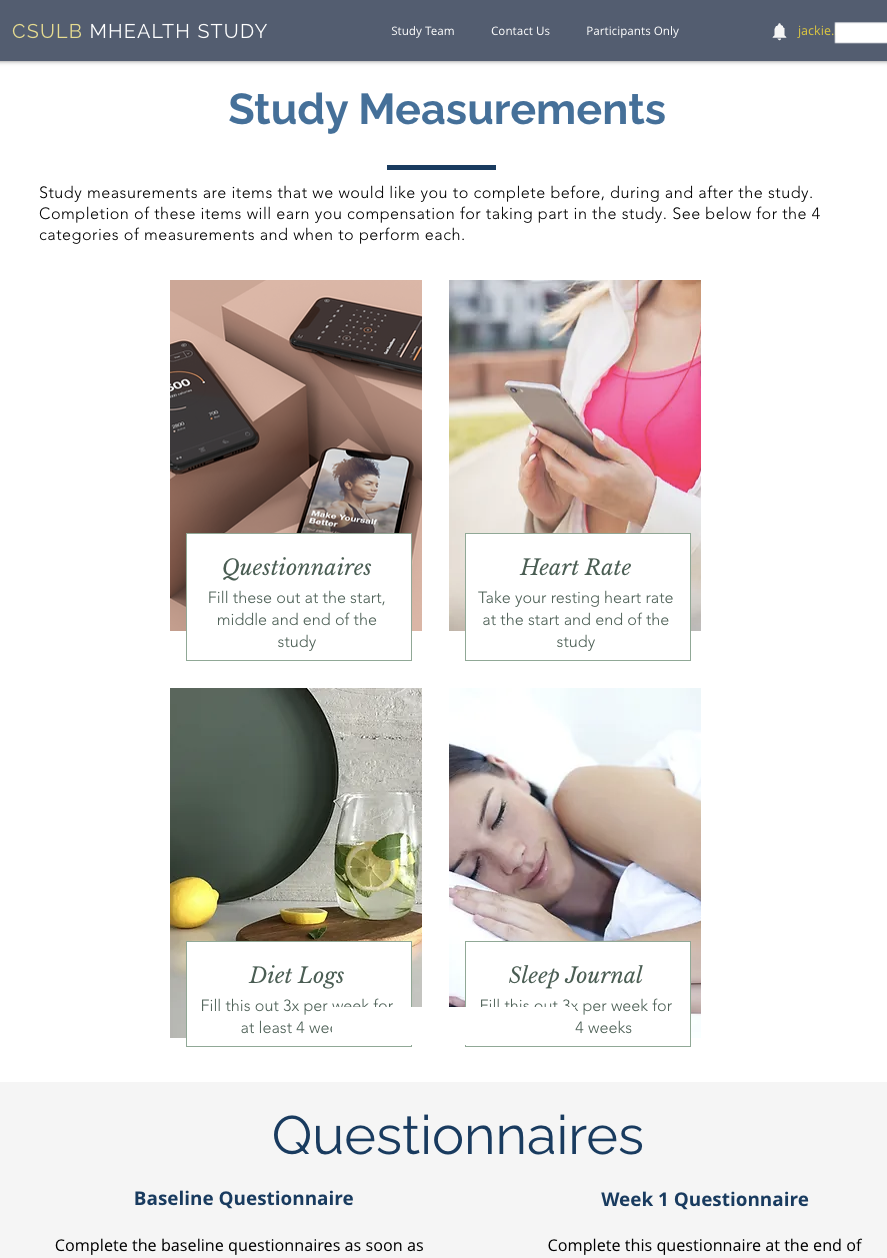


1. Getting Started Video Tutorials - Accessed by Level 1, Level 2 and Level 3 participants


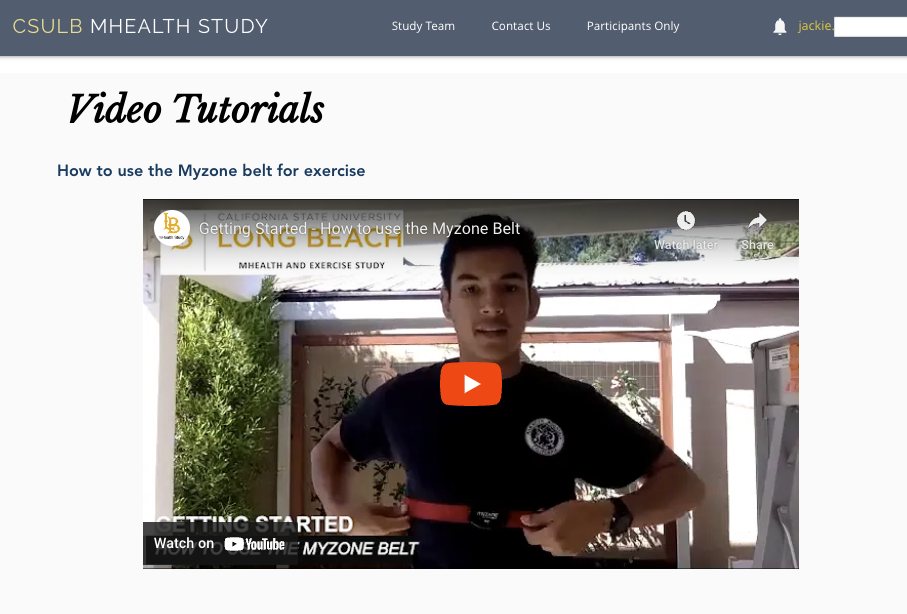


1. Wellness Education - Accessed by Level 1, Level 2 and Level 3 participants

Topics covered:

| Week | Topic | Resource |
| --- | --- | --- |
| 1 | Muscle-strengthening activities | Physical Activity Guidelines for Americans [24] |
| 2 | Aerobic activities | Physical Activity Guidelines for Americans [24] |
| 3 | Balance and flexibility activities | Physical Activity Guidelines for Americans [24] |
| 4 | Healthy eating patterns | Physical Activity Guidelines for Americans [24] |
| 5 | Myplate | Dietary Guidelines for Americans [25] |
| 6 | Exercise for mental health | Dietary Guidelines for Americans [25] |
| 7 | Sleep hygiene and mindfulness | Physical Activity Guidelines for Americans [24] |
| 8 | Establishing a consistent workout routine | Physical Activity Guidelines for Americans [24] |

Example page:


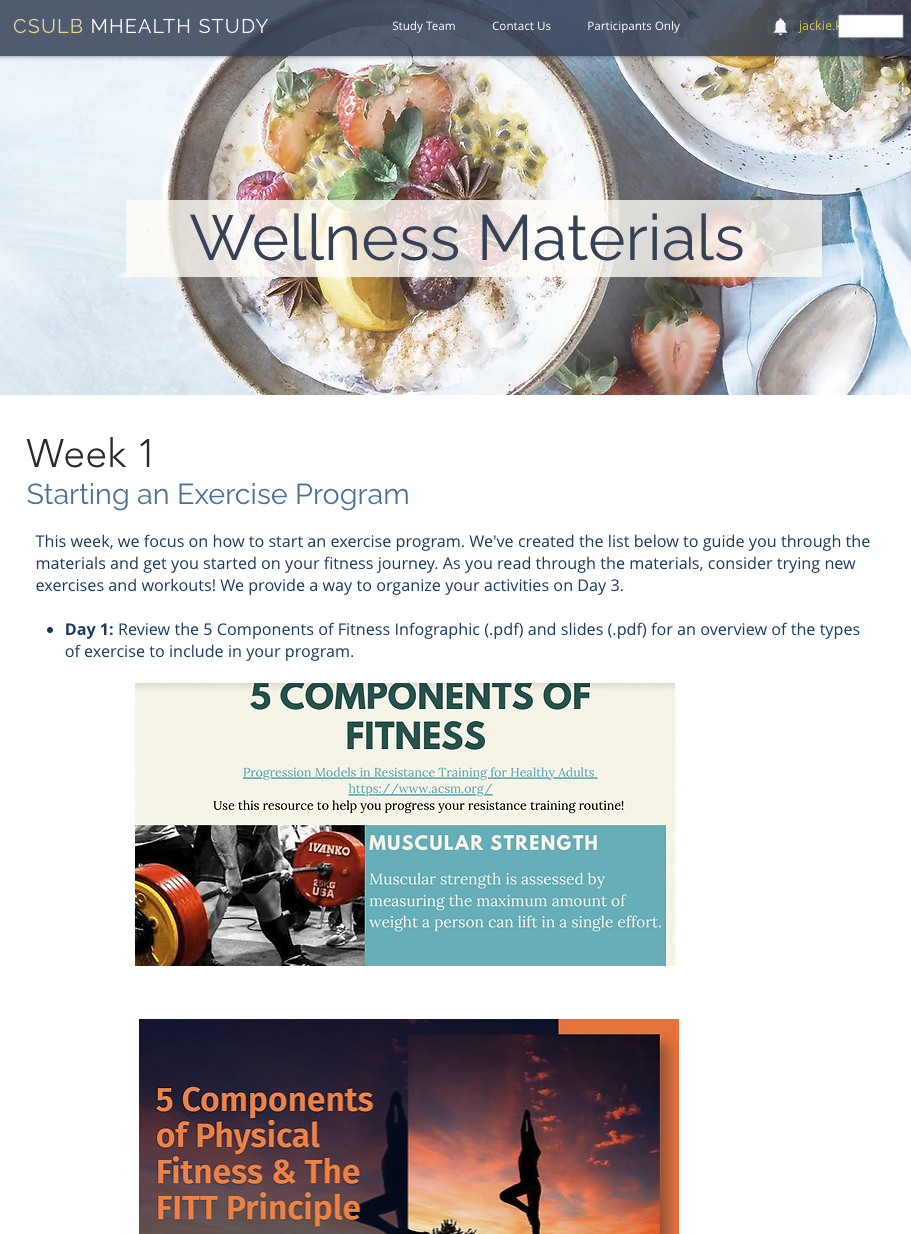


1. Exercise Videos Page – Accessed only by Level 2 participants


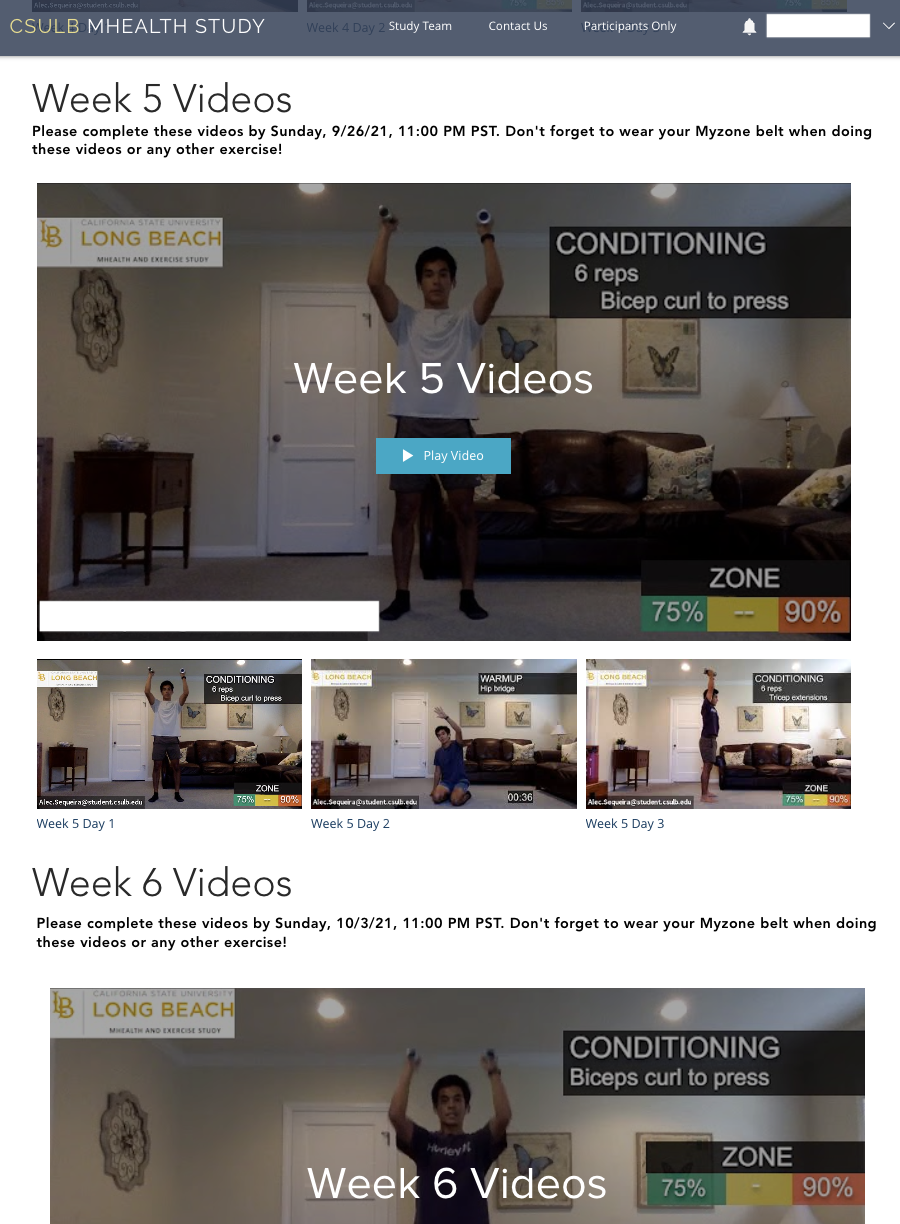


1. Exercise Class Schedule Page – Accessed only by Level 3 participants


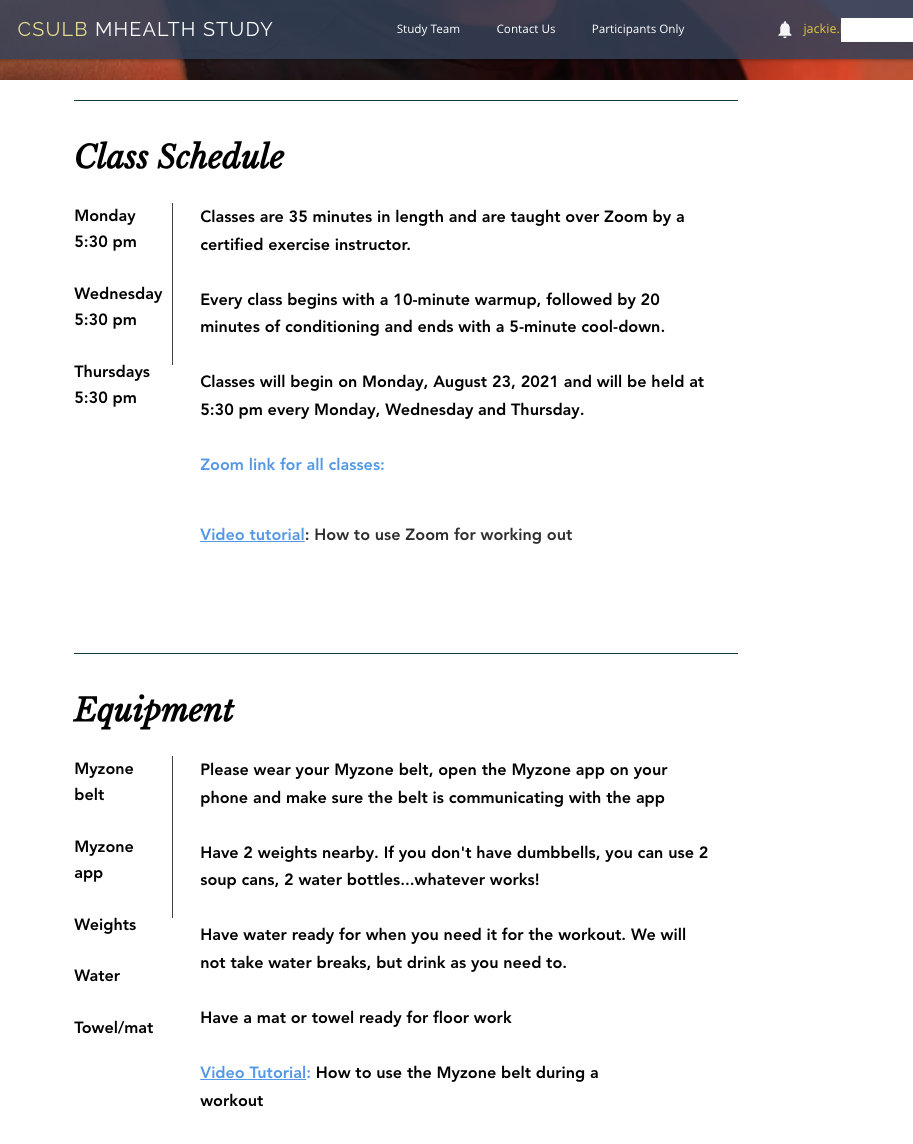

Supplement: Multimedia Appendix 1 [file formative_v8i1e52428_app1.docx]
