# Supplementary material for: Feasibility and Acceptability of a Mobile Health Exercise Intervention for Inactive Adults: 3-Arm Randomized Controlled Pilot Trial
Source: JMIR Form Res. 2024 Aug 9;8:e52428. doi: 10.2196/52428 (PMC11346126; doi:10.2196/52428)

**Multimedia Appendix 2. Emails**

Topics covered:

| Week | Topic | Resource |
| --- | --- | --- |
| 1 | Muscle-strengthening activities | Physical Activity Guidelines for Americans [24] |
| 2 | Aerobic activities | Physical Activity Guidelines for Americans [24] |
| 3 | Balance and flexibility activities | Physical Activity Guidelines for Americans [24] |
| 4 | Healthy eating patterns | Physical Activity Guidelines for Americans [24] |
| 5 | Myplate | Dietary Guidelines for Americans [25] |
| 6 | Exercise for mental health | Dietary Guidelines for Americans [25] |
| 7 | Sleep hygiene and mindfulness | Physical Activity Guidelines for Americans [24] |
| 8 | Establishing a consistent workout routine | Physical Activity Guidelines for Americans [24] |

Example Email: Week 7


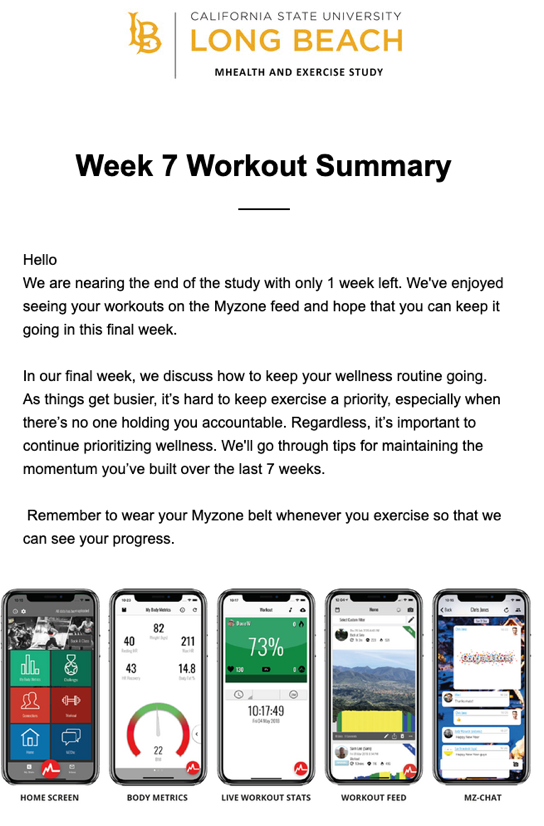


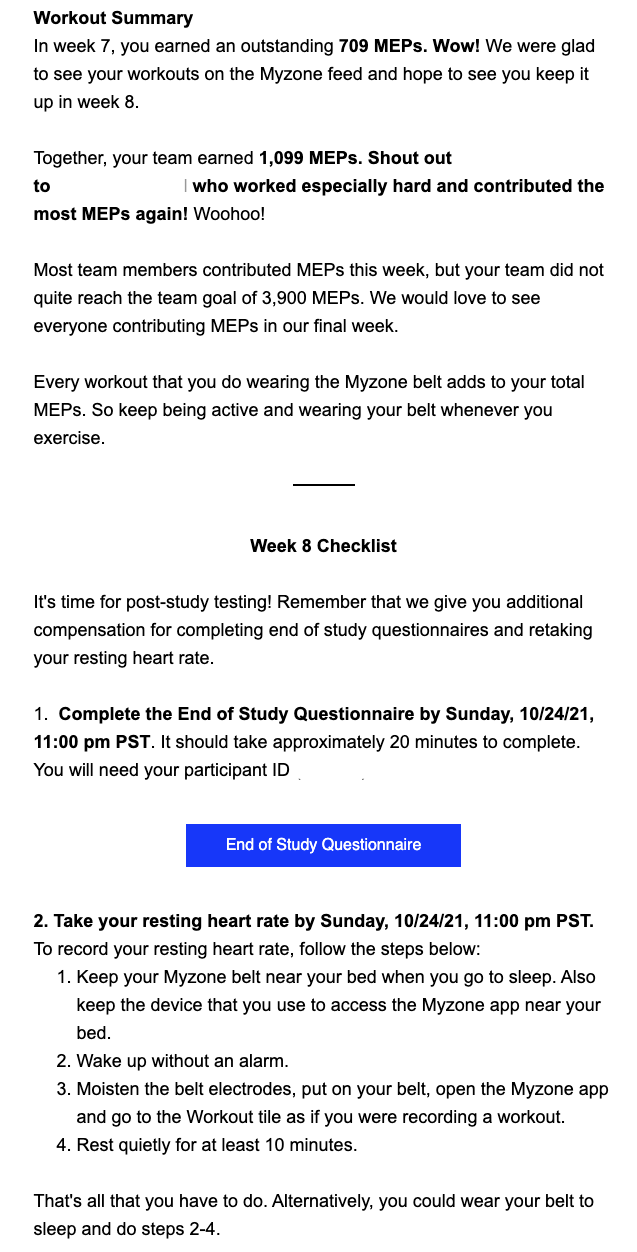


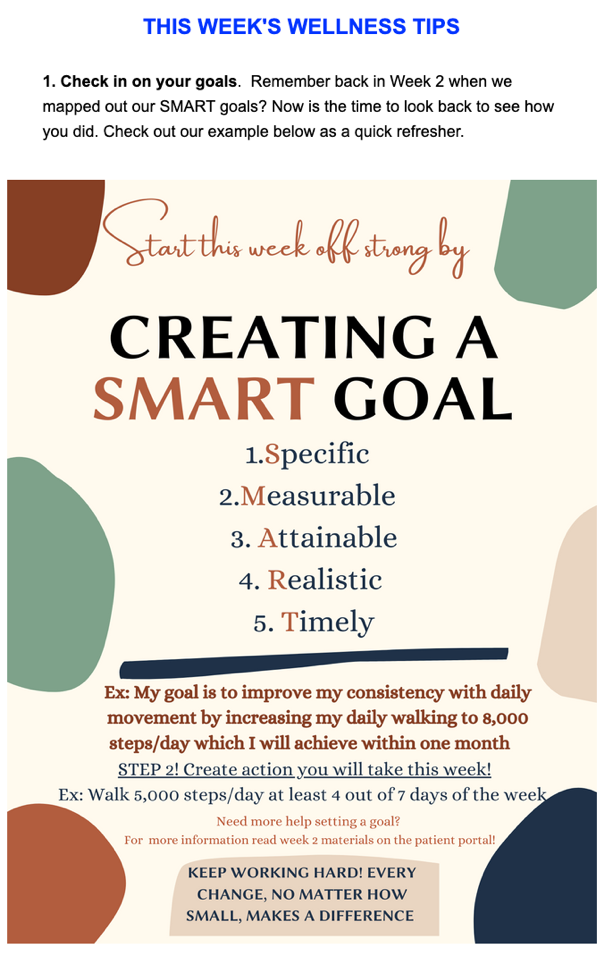


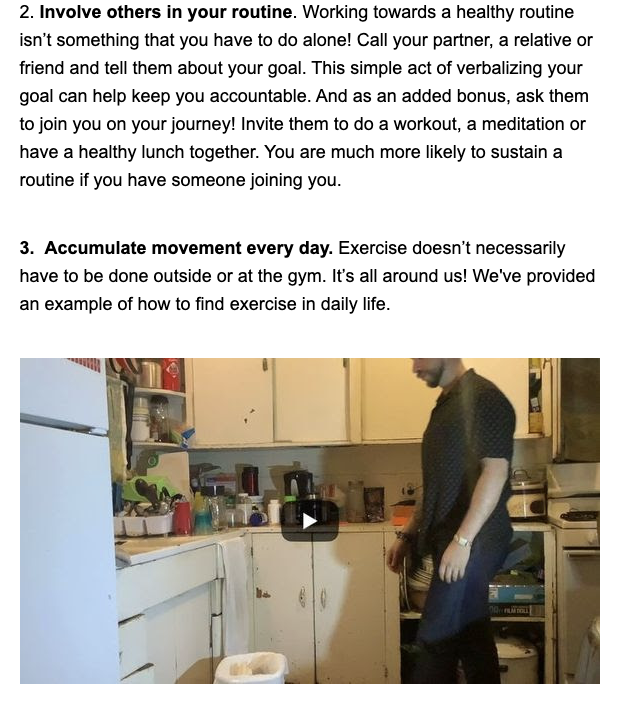

Supplement: Multimedia Appendix 2 [file formative_v8i1e52428_app2.docx]
