# Supplementary material for: Feasibility and Acceptability of a Mobile Health Exercise Intervention for Inactive Adults: 3-Arm Randomized Controlled Pilot Trial
Source: JMIR Form Res. 2024 Aug 9;8:e52428. doi: 10.2196/52428 (PMC11346126; doi:10.2196/52428)
Supplement: Multimedia Appendix 3 [file formative_v8i1e52428_app3.docx]

**Multimedia Appendix 3. Exercise Intervention**

Exercise Sessions for Weeks 1-2 (Focus: multi-joint exercise acclimation)

| **Dynamic Warmup (10 min, 50-60% maximal heart rate)** | | | |
| --- | --- | --- | --- |
| General – 2 min | Walk or jog in place | | |
| Sagittal – 1 min | March with knee holds | | |
| Sagittal – 1 min | Toe touch with chest opener | | |
| Frontal – 1 min | Side lunge with weight shift | | |
| Frontal – 1 min | Shuffle | | |
| Transverse – 1 min | Trunk rotation to knee | | |
| Transverse – 1 min | Split squat with rotation | | |
| Core activation – 1 min | Front plank hold | | |
| Core activation – 1 min | Hip bridge | | |
| **Conditioning (repeated for 3 rounds, total of 20 min, ≥70% HRmax)** | | | |
|  | Day 1 | Day 2 | Day 3 |
| Lower body sagittal – 1 min | Squats | Forward lunges | Deadlift |
| Upper body sagittal – 1 min | Overhead press | Front raise | Tricep extension |
| Lower body frontal – 1 min | Side lunge | Skater hops | Sumo deadlift |
| Upper body frontal – 1 min | Side raise | Upright row | Side bicep curl |
| Lower body transverse – 1 min | Curtsey lunge | Rear side lunge | 3-point lunge |
| Upper body transverse – 1 min | Pushup | Bent over row | Chest fly |
| Dynamic – 30 sec | Jumping Jacks | Burpee | Lawnmower row |
| **Cool Down (5 min, 60-70% maximal heart rate)** | | | |
| Core activation – 2 min | Front plank to side plank | | |
| Glute activation – 1 min | Hip bridge | | |
| **Flexibility (3 min)** | | | |
| Hip flexors/knee extensors – 1 min | Figure 4 stretch | | |
| Hip extensors/knee flexors – 1 min | Seated hamstring stretch | | |
| Pectoralis/Latissimus dorsi – 1 min | Kneeling chest and lat stretches | | |

Exercise Sessions for Weeks 3-4 (Focus: multi-joint and combination exercises)

| **Dynamic Warmup (10 min, 50-60% maximal heart rate) – Same as Weeks 1-2** | | | |
| --- | --- | --- | --- |
| **Conditioning (repeated for 3 rounds, total of 20 min, ≥75% HRmax)** | | | |
|  | Day 1 | Day 2 | Day 3 |
| Sagittal – 1 min | Squat + overhead press | Forward lunges | Deadlift |
| Sagittal – 1 min | Biceps curl | Front raise | Bicep curl + tricep extension |
| Frontal – 1 min | Side lunge | Skater hops | Sumo deadlift + upright row |
| Frontal – 1 min | Side raise | Side lunge + row | Side bicep curl |
| Transverse – 1 min | Curtsey lunge | Rear side lunge | 3-point lunge |
| Transverse – 1 min | Pushup | Bent over row | Pushup to T stand |
| Dynamic – 30 sec | Jumping Jacks | Burpee | Shuffle |
| **Cool Down (5 min, 60-70% maximal heart rate) – Same as Weeks 1-2** | | | |
| **Flexibility (3 min) – Same as Weeks 1-2** | | | |

Exercise Sessions for Weeks 5-6 (Focus: multi-joint and multi-planar exercises)

| **Dynamic Warmup (10 min, 50-60% maximal heart rate) – Same as Weeks 1-2** | | | |
| --- | --- | --- | --- |
| **Conditioning (repeated for 3 rounds, total of 20 min, ≥80% HRmax)** | | | |
|  | Day 1 | Day 2 | Day 3 |
| Sagittal – 1 min | Squat + overhead press | Forward lunges | Deadlift |
| Sagittal – 1 min | Split squat + bicep curl | Front raise | Bicep curl + tricep extension |
| Frontal – 1 min | Side lunge | Sumo deadlift + upright row | Side lunge |
| Frontal – 1 min | Side raise | Side lunge + bent over row | Lawnmower row |
| Transverse – 1 min | Curtsey + rear lunge | Rear side lunge | 3-point lunge |
| Transverse – 1 min | Pushup | Burpee | Pushup to T stand |
| Dynamic – 30 sec | Skater hop | Jumping jacks | Shuffle |
| **Cool Down (5 min, 60-70% maximal heart rate) – Same as Weeks 1-2** | | | |
| **Flexibility (3 min) – Same as Weeks 1-2** | | | |

Exercise Sessions for Weeks 7-8 (Focus: multi-joint, multi-planar exercises with ladder format)

| **Dynamic Warmup (10 min, 50-60% maximal heart rate) – Same as Weeks 1-2** | | | |
| --- | --- | --- | --- |
| **Conditioning (Total of 20 min, ≥85% HRmax)** | | | |
|  | Day 1 | Day 2 | Day 3 |
| Repetitions begin at 5 and increase by 5 every set until 20 min is reached, i.e. 5 reps, 10 reps, 15 reps, etc. | Squat + overhead press | Forward lunges | Deadlift |
|  | Split squat + bicep curl | Front raise | Bicep curl + tricep extension |
|  | Side lunge | Sumo deadlift + upright row | Side lunge |
|  | Side raise | Side lunge + bent over row | Lawnmower row |
|  | Curtsey + rear lunge | Rear side lunge | 3-point lunge |
|  | Pushup | Burpee | Pushup to T stand |
|  | Skater hop | Jumping jacks | Shuffle |
| **Cool Down (5 min, 60-70% maximal heart rate) – Same as Weeks 1-2** | | | |
| **Flexibility (3 min) – Same as Weeks 1-2** | | | |
