# Supplementary material for: Feasibility and Acceptability of a Mobile Health Exercise Intervention for Inactive Adults: 3-Arm Randomized Controlled Pilot Trial
Source: JMIR Form Res. 2024 Aug 9;8:e52428. doi: 10.2196/52428 (PMC11346126; doi:10.2196/52428)
Supplement: Multimedia Appendix 4 [file formative_v8i1e52428_app4.docx]

**Multimedia Appendix 4. User Experience Survey and Coding**

User Experience Survey

1. Which parts of the program did you like the most?
2. Which parts of the program did you like the least?
3. Which technology did you experience as easy?
4. Which technology did you experience as difficult?
5. What recommendations do you have for the program?

Response Coding

1. Most liked program components
   1. Coach’s exercise instruction
   2. Convenient workouts
   3. Social support from other participants
   4. Exercise format, variations and programming
   5. Physical activity awareness and accountability
2. Least liked program components
   1. No dislikes
   2. Schedule conflicts and time constraints
   3. Exercise format
   4. Myzone belt issues
   5. Repetitive exercises
3. Most user-friendly technological components
4. Least user-friendly technology components
5. Recommendations
   1. No recommendations
   2. More flexibility in workout schedule
   3. More methods of engaging with group members
   4. Incorporate input on music and exercise from participants
