# Supplementary material for: Feasibility and Acceptability of a Mobile Health Exercise Intervention for Inactive Adults: 3-Arm Randomized Controlled Pilot Trial
Source: JMIR Form Res. 2024 Aug 9;8:e52428. doi: 10.2196/52428 (PMC11346126; doi:10.2196/52428)
Supplement: Multimedia Appendix 5 [file formative_v8i1e52428_app5.pdf]

CONSORT-EHEALTH (V 1.6.1) - Submission/Publication Form

The CONSORT-EHEALTH checklist is intended for authors of randomized trials evaluating web-based and Internet-based applications/interventions, including mobile interventions, electronic games (incl multiplayer games), social media, certain telehealth applications, and other interactive and/or networked electronic applications. Some of the items (e.g. all subitems under item 5 - description of the intervention) may also be applicable for other study designs.

The goal of the CONSORT EHEALTH checklist and guideline is to be
a) a guide for reporting for authors of RCTs,
b) to form a basis for appraisal of an ehealth trial (in terms of validity)

CONSORT-EHEALTH items/subitems are MANDATORY reporting items for studies published in the Journal of Medical Internet Research and other journals / scientific societies endorsing the checklist.

Items numbered 1., 2., 3., 4a., 4b etc are original CONSORT or CONSORT-NPT (non-pharmacologic treatment) items.
Items with Roman numerals (i., ii, iii, iv etc.) are CONSORT-EHEALTH extensions/clarifications.

As the CONSORT-EHEALTH checklist is still considered in a formative stage, we would ask that you also RATE ON A SCALE OF 1-5 how important/useful you feel each item is FOR THE PURPOSE OF THE CHECKLIST and reporting guideline (optional).

Mandatory reporting items are marked with a red *.
In the textboxes, either copy & paste the relevant sections from your manuscript into this form - please include any quotes from your manuscript in QUOTATION MARKS,
or answer directly by providing additional information not in the manuscript, or elaborating on why the item was not relevant for this study.

YOUR ANSWERS WILL BE PUBLISHED AS A SUPPLEMENTARY FILE TO YOUR PUBLICATION IN JMIR AND ARE CONSIDERED PART OF YOUR PUBLICATION (IF ACCEPTED).
Please fill in these questions diligently. Information will not be copyedited, so please use proper spelling and grammar, use correct capitalization, and avoid abbreviations.

DO NOT FORGET TO SAVE AS PDF _AND_ CLICK THE SUBMIT BUTTON SO YOUR ANSWERS ARE IN OUR DATABASE !!!

Citation Suggestion (if you append the pdf as Appendix we suggest to cite this paper in the caption):
Eysenbach G, CONSORT-EHEALTH Group
CONSORT-EHEALTH: Improving and Standardizing Evaluation Reports of Web-based and Mobile Health Interventions
J Med Internet Res 2011;13(4):e126
URL: http://www.jmir.org/2011/4/e126/
doi: 10.2196/jmir.1923
PMID: 22209829

jackiedawsonphd@gmail.com Switch account

Not shared

Draft saved

* Indicates required question

Your name *

First Last

Jacqueline Dawson

Primary Affiliation (short), City, Country *

University of Toronto, Toronto, Canada

California State University, Long Beach

Your e-mail address *

abc@gmail.com

jacqueline.dawson@csulb.edu

Title of your manuscript *

Provide the (draft) title of your manuscript.

Feasibility and Acceptability of a Mobile Health Exercise Intervention for Inactive Adults: A Three-Arm Randomized Controlled Pilot Trial

Name of your App/Software/Intervention *

If there is a short and a long/alternate name, write the short name first and add the long name in brackets.

Videoconference group exercise

Evaluated Version (if any)

e.g. "V1", "Release 2017-03-01", "Version 2.0.27913"

Your answer

Language(s) *

What language is the intervention/app in? If multiple languages are available, separate by comma (e.g. "English, French")

English

URL of your Intervention Website or App

e.g. a direct link to the mobile app on app in appstore (itunes, Google Play), or URL of the website. If the intervention is a DVD or hardware, you can also link to an Amazon page.

Your answer

URL of an image/screenshot (optional)

Your answer

Accessibility *

Can an enduser access the intervention presently?

- ○ access is free and open
- ○ access only for special usergroups, not open
- ○ access is open to everyone, but requires payment/subscription/in-app purchases
- ◉ app/intervention no longer accessible
- ○ Other:

Primary Medical Indication/Disease/Condition *

e.g. "Stress", "Diabetes", or define the target group in brackets after the condition, e.g. "Autism (Parents of children with)", "Alzheimers (Informal Caregivers of)"

Inactive adults

Primary Outcomes measured in trial *

comma-separated list of primary outcomes reported in the trial

Feasibility, Acceptabilty

Secondary/other outcomes

Are there any other outcomes the intervention is expected to affect?

Resting heart rate, physical activity, quality of life, exercise dose

Recommended "Dose" *

What do the instructions for users say on how often the app should be used?

- ○ Approximately Daily
- ● Approximately Weekly
- ○ Approximately Monthly
- ○ Approximately Yearly
- ○ "as needed"
- ○ Other:

Approx. Percentage of Users (starters) still using the app as recommended after 3 months *

- (selected) unknown / not evaluated
- 0-10%
- 11-20%
- 21-30%
- 31-40%
- 41-50%
- 51-60%
- 61-70%
- 71%-80%
- 81-90%
- 91-100%
- Other:

Overall, was the app/intervention effective? *

- ○ yes: all primary outcomes were significantly better in intervention group vs control
- ◉ partly: SOME primary outcomes were significantly better in intervention group vs control
- ○ no statistically significant difference between control and intervention
- ○ potentially harmful: control was significantly better than intervention in one or more outcomes
- ○ inconclusive: more research is needed
- ○ Other:

Article Preparation Status/Stage *

At which stage in your article preparation are you currently (at the time you fill in this form)

- ○ not submitted yet - in early draft status
- ◉ not submitted yet - in late draft status, just before submission
- ○ submitted to a journal but not reviewed yet
- ○ submitted to a journal and after receiving initial reviewer comments
- ○ submitted to a journal and accepted, but not published yet
- ○ published
- ○ Other:

Journal *

If you already know where you will submit this paper (or if it is already submitted), please provide the journal name (if it is not JMIR, provide the journal name under "other")

- ○ not submitted yet / unclear where I will submit this
- ○ Journal of Medical Internet Research (JMIR)
- ◉ JMIR mHealth and UHealth
- ○ JMIR Serious Games
- ○ JMIR Mental Health
- ○ JMIR Public Health
- ○ JMIR Formative Research
- ○ Other JMIR sister journal
- ○ Other:

Is this a full powered effectiveness trial or a pilot/feasibility trial? *

- ◉ Pilot/feasibility
- ○ Fully powered

Manuscript tracking number *

If this is a JMIR submission, please provide the manuscript tracking number under "other" (The ms tracking number can be found in the submission acknowledgement email, or when you login as author in JMIR. If the paper is already published in JMIR, then the ms tracking number is the four-digit number at the end of the DOI, to be found at the bottom of each published article in JMIR)

- (selected) no ms number (yet) / not (yet) submitted to / published in JMIR
- Other:

TITLE AND ABSTRACT

1a) TITLE: Identification as a randomized trial in the title

1a) Does your paper address CONSORT item 1a? *

I.e does the title contain the phrase "Randomized Controlled Trial"? (if not, explain the reason under "other")

- (selected) yes
- Other:

1a-i) Identify the mode of delivery in the title

Identify the mode of delivery. Preferably use “web-based” and/or “mobile” and/or “electronic game” in the title. Avoid ambiguous terms like “online”, “virtual”, “interactive”. Use “Internet-based” only if Intervention includes non-web-based Internet components (e.g. email), use “computer-based” or “electronic” only if offline products are used. Use “virtual” only in the context of “virtual reality” (3-D worlds). Use “online” only in the context of “online support groups”. Complement or substitute product names with broader terms for the class of products (such as “mobile” or “smart phone” instead of “iphone”), especially if the application runs on different platforms.

	1	2	3	4	5	
subitem not at all important	○	○	○	○	◉	essential

Clear selection

Does your paper address subitem 1a-i? *

Copy and paste relevant sections from manuscript title (include quotes in quotation marks "like this" to indicate direct quotes from your manuscript), or elaborate on this item by providing additional information not in the ms, or briefly explain why the item is not applicable/relevant for your study

Title contains a specific mode of delivery: "Feasibility and Acceptability of a Mobile Health Exercise Intervention for Inactive Adults: A Three-Arm Randomized Controlled Pilot Trial"

1a-ii) Non-web-based components or important co-interventions in title

Mention non-web-based components or important co-interventions in title, if any (e.g., “with telephone support”).

	1	2	3	4	5	
subitem not at all important	○	○	○	◉	○	essential

Clear selection

Does your paper address subitem 1a-ii?

Copy and paste relevant sections from manuscript title (include quotes in quotation marks "like this" to indicate direct quotes from your manuscript), or elaborate on this item by providing additional information not in the ms, or briefly explain why the item is not applicable/relevant for your study

Title does not contain other intervention arms, but this is described throughout the manuscript. For example, in the abstract, the interventions are mentioned: "This study aimed to determine the feasibility and acceptability of an mHealth exercise intervention delivered in 3 ways: health education emails, asynchronous exercise videos or synchronous videoconference exercise classes."

1a-iii) Primary condition or target group in the title

Mention primary condition or target group in the title, if any (e.g., “for children with Type I Diabetes”) Example: A Web-based and Mobile Intervention with Telephone Support for Children with Type I Diabetes: Randomized Controlled Trial

	1	2	3	4	5	
subitem not at all important	○	○	○	○	●	essential

Clear selection

Does your paper address subitem 1a-iii? *

Copy and paste relevant sections from manuscript title (include quotes in quotation marks "like this" to indicate direct quotes from your manuscript), or elaborate on this item by providing additional information not in the ms, or briefly explain why the item is not applicable/relevant for your study

Title contains target group: "Feasibility and Acceptability of a Mobile Health Exercise Intervention for Inactive Adults: A Three-Arm Randomized Controlled Pilot Trial"

1b) ABSTRACT: Structured summary of trial design, methods, results, and conclusions

NPT extension: Description of experimental treatment, comparator, care providers, centers, and blinding status.

1b-i) Key features/functionalities/components of the intervention and comparator in the METHODS section of the ABSTRACT

Mention key features/functionalities/components of the intervention and comparator in the abstract. If possible, also mention theories and principles used for designing the site. Keep in mind the needs of systematic reviewers and indexers by including important synonyms. (Note: Only report in the abstract what the main paper is reporting. If this information is missing from the main body of text, consider adding it)

	1	2	3	4	5	
subitem not at all important	○	○	○	○	◉	essential

Clear selection

Does your paper address subitem 1b-i? *

Copy and paste relevant sections from the manuscript abstract (include quotes in quotation marks "like this" to indicate direct quotes from your manuscript), or elaborate on this item by providing additional information not in the ms, or briefly explain why the item is not applicable/relevant for your study

Yes. Abstract contains key features of the intervention groups: "8 weeks of increasing PA behavioral support: Level 1 (health education + objective monitoring, n=26), Level 2 (Level 1 + pre-recorded exercise videos, n=30), or Level 3 (Level 1 + videoconference group exercise with a live instructor, n=28)."

1b-ii) Level of human involvement in the METHODS section of the ABSTRACT

Clarify the level of human involvement in the abstract, e.g., use phrases like “fully automated” vs. “therapist/nurse/care provider/physician-assisted” (mention number and expertise of providers involved, if any). (Note: Only report in the abstract what the main paper is reporting. If this information is missing from the main body of text, consider adding it)

	1	2	3	4	5	
subitem not at all important	○	○	○	○	◉	essential

Clear selection

Does your paper address subitem 1b-ii?

Copy and paste relevant sections from the manuscript abstract (include quotes in quotation marks "like this" to indicate direct quotes from your manuscript), or elaborate on this item by providing additional information not in the ms, or briefly explain why the item is not applicable/relevant for your study

Yes. Abstract mentions whether the intervention was synchronous, asynchronous or simply monitoring. "mHealth exercise intervention delivered in 3 ways: health education emails, asynchronous exercise videos or synchronous videoconference exercise classes."; "Level 1 (health education + objective monitoring, n=26), Level 2 (Level 1 + pre-recorded exercise videos, n=30), or Level 3 (Level 1 + videoconference group exercise with a live instructor, n=28). "

1b-iii) Open vs. closed, web-based (self-assessment) vs. face-to-face assessments in the METHODS section of the ABSTRACT

Mention how participants were recruited (online vs. offline), e.g., from an open access website or from a clinic or a closed online user group (closed usergroup trial), and clarify if this was a purely web-based trial, or there were face-to-face components (as part of the intervention or for assessment). Clearly say if outcomes were self-assessed through questionnaires (as common in web-based trials). Note: In traditional offline trials, an open trial (open-label trial) is a type of clinical trial in which both the researchers and participants know which treatment is being administered. To avoid confusion, use “blinded” or “unblinded” to indicated the level of blinding instead of “open”, as “open” in web-based trials usually refers to “open access” (i.e. participants can self-enrol). (Note: Only report in the abstract what the main paper is reporting. If this information is missing from the main body of text, consider adding it)

	1	2	3	4	5	
subitem not at all important	○	○	○	◉	○	essential

Clear selection

Does your paper address subitem 1b-iii?

Copy and paste relevant sections from the manuscript abstract (include quotes in quotation marks "like this" to indicate direct quotes from your manuscript), or elaborate on this item by providing additional information not in the ms, or briefly explain why the item is not applicable/relevant for your study

Yes. Recruitment is mentioned as occuring online: "Physically inactive (< 30 min/week of exercise) adults (females 31.5 ± 11.3 yr, males 34.1 ± 28.9 yr, mean ± SD) were recruited online " Outcomes are mentioned as being self-assessed. "Secondary outcomes were self-assessed at baseline and 8 weeks and included resting heart rate, PA and quality of life questionnaires, while exercise dose was evaluated throughout the intervention."

1b-iv) RESULTS section in abstract must contain use data

Report number of participants enrolled/assessed in each group, the use/uptake of the intervention (e.g., attrition/adherence metrics, use over time, number of logins etc.), in addition to primary/secondary outcomes. (Note: Only report in the abstract what the main paper is reporting. If this information is missing from the main body of text, consider adding it)

	1	2	3	4	5	
subitem not at all important	○	○	○	○	◉	essential

Clear selection

Does your paper address subitem 1b-iv?

Copy and paste relevant sections from the manuscript abstract (include quotes in quotation marks "like this" to indicate direct quotes from your manuscript), or elaborate on this item by providing additional information not in the ms, or briefly explain why the item is not applicable/relevant for your study

Yes. Number of participants, adherence, retention and satisfaction are reported. "Between August 2020 and August 2021, 204 adults were screened for eligibility. Out of 135 eligible participants, 84 (62%) enrolled in the study. Retention was 50% (13/26) in Level 1, 60% (18/30) in Level 2 and 82% (23/28) in Level 3, while adherence was 31% (8/26) in Level 1, 40% (12/30) in Level 2 and 75% (21/28) in Level 3. Program satisfaction was highest in participants receiving exercise videos (90%, Level 2) or exercise classes (80%, Level 3), while only 63% of Level 1 reported the program as enjoyable"

1b-v) CONCLUSIONS/DISCUSSION in abstract for negative trials

Conclusions/Discussions in abstract for negative trials: Discuss the primary outcome - if the trial is negative (primary outcome not changed), and the intervention was not used, discuss whether negative results are attributable to lack of uptake and discuss reasons. (Note: Only report in the abstract what the main paper is reporting. If this information is missing from the main body of text, consider adding it)

	1	2	3	4	5	
subitem not at all important	○	○	○	◉	○	essential

Clear selection

Does your paper address subitem 1b-v?

Copy and paste relevant sections from the manuscript abstract (include quotes in quotation marks "like this" to indicate direct quotes from your manuscript), or elaborate on this item by providing additional information not in the ms, or briefly explain why the item is not applicable/relevant for your study

Yes. One of the study arms was not deemed feasible and this was mentioned in the conclusion. "Only the videoconference exercise classes met feasibility criteria, although both videoconference and pre-recorded videos had good acceptability"

INTRODUCTION

2a) In INTRODUCTION: Scientific background and explanation of rationale

2a-i) Problem and the type of system/solution

Describe the problem and the type of system/solution that is object of the study: intended as stand-alone intervention vs. incorporated in broader health care program? Intended for a particular patient population? Goals of the intervention, e.g., being more cost-effective to other interventions, replace or complement other solutions? (Note: Details about the intervention are provided in “Methods” under 5)

	1	2	3	4	5	
subitem not at all important	○	○	○	○	◉	essential

Clear selection

Does your paper address subitem 2a-i? *

Copy and paste relevant sections from the manuscript (include quotes in quotation marks "like this" to indicate direct quotes from your manuscript), or elaborate on this item by providing additional information not in the ms, or briefly explain why the item is not applicable/relevant for your study

Yes. This intervention evaluates the feasibility of videoconference group exercise compared to previously used interventions. "Given the high rates of participant dropout, poor adherence or missing outcome data reported in previous synchronous mHealth investigations (13, 17, 18), more information on the feasibility and acceptability of mHealth interventions with videoconferencing is needed. "

2a-ii) Scientific background, rationale: What is known about the (type of) system

Scientific background, rationale: What is known about the (type of) system that is the object of the study (be sure to discuss the use of similar systems for other conditions/diagnoses, if appropiate), motivation for the study, i.e. what are the reasons for and what is the context for this specific study, from which stakeholder viewpoint is the study performed, potential impact of findings [2]. Briefly justify the choice of the comparator.

	1	2	3	4	5	
subitem not at all important	○	○	○	○	◉	essential

Clear selection

Does your paper address subitem 2a-ii? *

Copy and paste relevant sections from the manuscript (include quotes in quotation marks "like this" to indicate direct quotes from your manuscript), or elaborate on this item by providing additional information not in the ms, or briefly explain why the item is not applicable/relevant for your study

Yes. Prior investigations using asynchronous exercise and objective monitoring are discussed in the Introduction. "Previous mHealth exercise studies have predominantly used objective monitoring alone or in combination with asynchronous interventions to promote exercise-related behaviors, including PA participation and adherence, overall PA time, weekly moderate-to-vigorous intensity physical activity (MVPA) time, walking time and heart rate-based exercise dose (5, 8, 9). Yet, several systematic reviews have found no evidence of objective monitoring increasing PA, even when combined with health education materials, as minimal contact interventions are not effective at improving participation or adherence (5, 8, 9). When additional behavioral support through asynchronous components, such as self-directed website or mobile app use, are combined with objective monitoring, mHealth exercise interventions are slightly more effective at increasing adherence and eliciting PA behavior change (8, 9), although the greatest increases in PA have been observed when interventions include a synchronous component such as text messaging or phone call with study personnel (8, 9). "

2b) In INTRODUCTION: Specific objectives or hypotheses

Does your paper address CONSORT subitem 2b? *

Copy and paste relevant sections from the manuscript (include quotes in quotation marks "like this" to indicate direct quotes from your manuscript), or elaborate on this item by providing additional information not in the ms, or briefly explain why the item is not applicable/relevant for your study

Yes. Primary and secondary aims are described. "Therefore, the primary aim of this study was to determine the feasibility and acceptability of an 8-week mHealth progressive exercise intervention for physically inactive adults, designed as a pilot randomized controlled trial (RCT) with 3 levels of behavioral support. A secondary aim explored changes in resting heart rate, compliance through exercise dose, self-reported PA and quality of life. "

METHODS

3a) Description of trial design (such as parallel, factorial) including allocation ratio

Does your paper address CONSORT subitem 3a? *

Copy and paste relevant sections from the manuscript (include quotes in quotation marks "like this" to indicate direct quotes from your manuscript), or elaborate on this item by providing additional information not in the ms, or briefly explain why the item is not applicable/relevant for your study

Yes. "We conducted a 3-arm randomized pilot trial of an 8-week intervention for physically inactive adults (ClinicalTrials.gov NCT05192421) that randomized participants (1:1:1) to increasing levels of behavioral support (Level 1 – lifestyle education + objective monitoring, Level 2 – asynchronous exercise videos + lifestyle education + objective monitoring, Level 3 – synchronous videoconference exercise classes + lifestyle education + objective monitoring). "

3b) Important changes to methods after trial commencement (such as eligibility criteria), with reasons

Does your paper address CONSORT subitem 3b? *

Copy and paste relevant sections from the manuscript (include quotes in quotation marks "like this" to indicate direct quotes from your manuscript), or elaborate on this item by providing additional information not in the ms, or briefly explain why the item is not applicable/relevant for your study

No. Methods were not changed after trial commencement.

3b-i) Bug fixes, Downtimes, Content Changes

Bug fixes, Downtimes, Content Changes: ehealth systems are often dynamic systems. A description of changes to methods therefore also includes important changes made on the intervention or comparator during the trial (e.g., major bug fixes or changes in the functionality or content) (5-iii) and other “unexpected events” that may have influenced study design such as staff changes, system failures/downtimes, etc. [2].

	1	2	3	4	5	
subitem not at all important	○	◉	○	○	○	essential

Clear selection

Does your paper address subitem 3b-i?

Copy and paste relevant sections from the manuscript (include quotes in quotation marks "like this" to indicate direct quotes from your manuscript), or elaborate on this item by providing additional information not in the ms, or briefly explain why the item is not applicable/relevant for your study

No. There were no bug fixes or content changes to the mobile app while the study was being conducted.

4a) Eligibility criteria for participants

Does your paper address CONSORT subitem 4a? *

Copy and paste relevant sections from the manuscript (include quotes in quotation marks "like this" to indicate direct quotes from your manuscript), or elaborate on this item by providing additional information not in the ms, or briefly explain why the item is not applicable/relevant for your study

Yes. "Inclusion criteria included physically inactive adults (<60 min of moderate-to-vigorous physical activity per week) aged ≥18 years, who possessed a mobile device with high-speed internet access and the ability to use the device and understand English. Potential participants were recruited through Facebook and Instagram social media posts and screened for eligibility using a questionnaire to assess age and internet access, and the International Physical Activity Questionnaire (20) to assess PA levels. Potential contraindications to exercise were assessed using the American College of Sports Medicine exercise preparticipation questionnaire (21). Exclusion criteria included history of unstable cardiac or pulmonary disease, current use of tricyclic antidepressant or clozapine medications, or orthopedic issues that impaired walking. "

4a-i) Computer / Internet literacy

Computer / Internet literacy is often an implicit “de facto” eligibility criterion - this should be explicitly clarified.

	1	2	3	4	5	
subitem not at all important	○	○	○	◉	○	essential

Clear selection

Does your paper address subitem 4a-i?

Copy and paste relevant sections from the manuscript (include quotes in quotation marks "like this" to indicate direct quotes from your manuscript), or elaborate on this item by providing additional information not in the ms, or briefly explain why the item is not applicable/relevant for your study

Yes. "Inclusion criteria included physically inactive adults (<60 min of moderate-to-vigorous physical activity per week) aged 18 years, who possessed a mobile device with high-speed internet access and the ability to use the device and understand English."

4a-ii) Open vs. closed, web-based vs. face-to-face assessments:

Open vs. closed, web-based vs. face-to-face assessments: Mention how participants were recruited (online vs. offline), e.g., from an open access website or from a clinic, and clarify if this was a purely web-based trial, or there were face-to-face components (as part of the intervention or for assessment), i.e., to what degree got the study team to know the participant. In online-only trials, clarify if participants were quasi-anonymous and whether having multiple identities was possible or whether technical or logistical measures (e.g., cookies, email confirmation, phone calls) were used to detect/prevent these.

	1	2	3	4	5	
subitem not at all important	○	○	○	◉	○	essential

Clear selection

Does your paper address subitem 4a-ii? *

Copy and paste relevant sections from the manuscript (include quotes in quotation marks "like this" to indicate direct quotes from your manuscript), or elaborate on this item by providing additional information not in the ms, or briefly explain why the item is not applicable/relevant for your study

Yes, both recruitment and assessments were web-based. "Potential participants were recruited through Facebook and Instagram social media posts and screened for eligibility using a questionnaire to assess age and internet access, and the International Physical Activity Questionnaire (20) to assess PA levels. " "All measurements were self-assessed remotely by participants at baseline and post-intervention except for exercise dose, which was monitored remotely and continuously throughout the intervention."

4a-iii) Information giving during recruitment

Information given during recruitment. Specify how participants were briefed for recruitment and in the informed consent procedures (e.g., publish the informed consent documentation as appendix, see also item X26), as this information may have an effect on user self-selection, user expectation and may also bias results.

	1	2	3	4	5	
subitem not at all important	○	○	○	◉	○	essential

Clear selection

Does your paper address subitem 4a-iii?

Copy and paste relevant sections from the manuscript (include quotes in quotation marks "like this" to indicate direct quotes from your manuscript), or elaborate on this item by providing additional information not in the ms, or briefly explain why the item is not applicable/relevant for your study

Yes. In the informed consent, participants were told about the randomization, each study group, the intervention, assessments and where to seek help if injured. "Participants were told that they would be randomly assigned to receive certain resources but were unaware of what was received relative to other study arms."

4b) Settings and locations where the data were collected

Does your paper address CONSORT subitem 4b? *

Copy and paste relevant sections from the manuscript (include quotes in quotation marks "like this" to indicate direct quotes from your manuscript), or elaborate on this item by providing additional information not in the ms, or briefly explain why the item is not applicable/relevant for your study

Yes. "All measurements were self-assessed remotely by participants at baseline and post-intervention except for exercise dose, which was monitored remotely and continuously throughout the intervention."

4b-i) Report if outcomes were (self-)assessed through online questionnaires

Clearly report if outcomes were (self-)assessed through online questionnaires (as common in web-based trials) or otherwise.

	1	2	3	4	5	
subitem not at all important	○	○	○	○	◉	essential

Clear selection

Does your paper address subitem 4b-i? *

Copy and paste relevant sections from the manuscript (include quotes in quotation marks "like this" to indicate direct quotes from your manuscript), or elaborate on this item by providing additional information not in the ms, or briefly explain why the item is not applicable/relevant for your study

Yes, certain outcomes were assessed through questionnaires. "The IPAQ was administered remotely using an online survey application (Qualtrics, Seattle, USA)." "QOL was assessed through the Short Form-36 (SF-36) (29) and administered remotely using an online survey application (Qualtrics). "

4b-ii) Report how institutional affiliations are displayed

Report how institutional affiliations are displayed to potential participants [on ehealth media], as affiliations with prestigious hospitals or universities may affect volunteer rates, use, and reactions with regards to an intervention.(Not a required item – describe only if this may bias results)

	1	2	3	4	5	
subitem not at all important	○	○	◉	○	○	essential

Clear selection

Does your paper address subitem 4b-ii?

Copy and paste relevant sections from the manuscript (include quotes in quotation marks "like this" to indicate direct quotes from your manuscript), or elaborate on this item by providing additional information not in the ms, or briefly explain why the item is not applicable/relevant for your study

Yes, affiliation is mentioned in the Methods and sample pages from the study website are shown in the Multimedia Appendix. "The study website listed affiliation with the investigators' university, contained all resources referenced in the weekly email and was tailored to each participant's group assignment. "

5) The interventions for each group with sufficient details to allow replication, including how and when they were actually administered

5-i) Mention names, credential, affiliations of the developers, sponsors, and owners

Mention names, credential, affiliations of the developers, sponsors, and owners [6] (if authors/evaluators are owners or developer of the software, this needs to be declared in a “Conflict of interest” section or mentioned elsewhere in the manuscript).

	1	2	3	4	5	
subitem not at all important	○	○	○	○	◉	essential

Clear selection

Does your paper address subitem 5-i?

Copy and paste relevant sections from the manuscript (include quotes in quotation marks "like this" to indicate direct quotes from your manuscript), or elaborate on this item by providing additional information not in the ms, or briefly explain why the item is not applicable/relevant for your study

Yes. Names and affiliations of the authors are listed on the title page. "Jacqueline K Dawson1, PhD, Alison Ede2, PhD, Madeleine Phan1, Alec Sequeira2, Hsiang-Ling Teng1, PT, PhD, Ayla Donlin3, EdD

1Department of Physical Therapy, California State University, Long Beach
2Department of Kinesiology, California State University, Long Beach
3LifeFit Center, California State University, Long Beach"

5-ii) Describe the history/development process

Describe the history/development process of the application and previous formative evaluations (e.g., focus groups, usability testing), as these will have an impact on adoption/use rates and help with interpreting results.

	1	2	3	4	5	
subitem not at all important	○	○	○	●	○	essential

Clear selection

Does your paper address subitem 5-ii?

Copy and paste relevant sections from the manuscript (include quotes in quotation marks "like this" to indicate direct quotes from your manuscript), or elaborate on this item by providing additional information not in the ms, or briefly explain why the item is not applicable/relevant for your study

No. This study did not use a formative evaluation to inform the intervention. However, that is a recommendation that is made in the discussion for future work. "Given that the unsupervised, asynchronous format is a popular format used in healthcare settings for preventative and outpatient rehabilitative programs, future studies that incorporate asynchronous delivery should first employ formative approaches, such as usability testing or community-based participatory research, to inform the development of the intervention with user feedback. "

5-iii) Revisions and updating

Revisions and updating. Clearly mention the date and/or version number of the application/intervention (and comparator, if applicable) evaluated, or describe whether the intervention underwent major changes during the evaluation process, or whether the development and/or content was “frozen” during the trial. Describe dynamic components such as news feeds or changing content which may have an impact on the replicability of the intervention (for unexpected events see item 3b).

	1	2	3	4	5	
subitem not at all important	○	◉	○	○	○	essential

Clear selection

Does your paper address subitem 5-iii?

Copy and paste relevant sections from the manuscript (include quotes in quotation marks "like this" to indicate direct quotes from your manuscript), or elaborate on this item by providing additional information not in the ms, or briefly explain why the item is not applicable/relevant for your study

No. The application/intervention did not change during the trial.

5-iv) Quality assurance methods

Provide information on quality assurance methods to ensure accuracy and quality of information provided [1], if applicable.

	1	2	3	4	5	
subitem not at all important	○	○	◉	○	○	essential

Clear selection

Does your paper address subitem 5-iv?

Copy and paste relevant sections from the manuscript (include quotes in quotation marks "like this" to indicate direct quotes from your manuscript), or elaborate on this item by providing additional information not in the ms, or briefly explain why the item is not applicable/relevant for your study

No. Quality assurance methods were not employed.

5-v) Ensure replicability by publishing the source code, and/or providing screenshots/screen-capture video, and/or providing flowcharts of the algorithms used

Ensure replicability by publishing the source code, and/or providing screenshots/screen-capture video, and/or providing flowcharts of the algorithms used. Replicability (i.e., other researchers should in principle be able to replicate the study) is a hallmark of scientific reporting.

	1	2	3	4	5	
subitem not at all important	○	○	○	○	◉	essential

Clear selection

Does your paper address subitem 5-v?

Copy and paste relevant sections from the manuscript (include quotes in quotation marks "like this" to indicate direct quotes from your manuscript), or elaborate on this item by providing additional information not in the ms, or briefly explain why the item is not applicable/relevant for your study

Yes. Screenshots of the website, mobile app and weekly emails are provided in the Multimedia appendix.

5-vi) Digital preservation

Digital preservation: Provide the URL of the application, but as the intervention is likely to change or disappear over the course of the years; also make sure the intervention is archived (Internet Archive, webcitation.org, and/or publishing the source code or screenshots/videos alongside the article). As pages behind login screens cannot be archived, consider creating demo pages which are accessible without login.

	1	2	3	4	5	
subitem not at all important	○	○	◉	○	○	essential

Clear selection

Does your paper address subitem 5-vi?

Copy and paste relevant sections from the manuscript (include quotes in quotation marks "like this" to indicate direct quotes from your manuscript), or elaborate on this item by providing additional information not in the ms, or briefly explain why the item is not applicable/relevant for your study

The website used in the intervention is mentioned in the manuscript and screenshots of the pages are provided in the Multimedia Appendix. "Participants accessed the study website (https://www.csulbmhealthstudy.com/) with a username and password that were provided following orientation to the MZ-3 and Myzone app."

5-vii) Access

Access: Describe how participants accessed the application, in what setting/context, if they had to pay (or were paid) or not, whether they had to be a member of specific group. If known, describe how participants obtained "access to the platform and Internet" [1]. To ensure access for editors/reviewers/readers, consider to provide a "backdoor" login account or demo mode for reviewers/readers to explore the application (also important for archiving purposes, see vi).

	1	2	3	4	5	
subitem not at all important	○	○	○	○	◉	essential

Clear selection

Does your paper address subitem 5-vii? *

Copy and paste relevant sections from the manuscript (include quotes in quotation marks "like this" to indicate direct quotes from your manuscript), or elaborate on this item by providing additional information not in the ms, or briefly explain why the item is not applicable/relevant for your study

Participants were provided with free accounts to access the study website and Myzone app during the study. "Participants accessed the study website (https://www.csulbmhealthstudy.com/) with a username and password that were provided following orientation to the MZ-3 and Myzone app. " "All participants were provided a heart rate (HR) monitoring device and access to a mobile app (Myzone MZ-3, Chicago, IL) to track exercise intensity and were encouraged to exercise at least 3 sessions/week while wearing the device. "

5-viii) Mode of delivery, features/functionalities/components of the intervention and comparator, and the theoretical framework

Describe mode of delivery, features/functionalities/components of the intervention and comparator, and the theoretical framework [6] used to design them (instructional strategy [1], behaviour change techniques, persuasive features, etc., see e.g., [7, 8] for terminology). This includes an in-depth description of the content (including where it is coming from and who developed it) [1]," whether [and how] it is tailored to individual circumstances and allows users to track their progress and receive feedback" [6]. This also includes a description of communication delivery channels and – if computer-mediated communication is a component – whether communication was synchronous or asynchronous [6]. It also includes information on presentation strategies [1], including page design principles, average amount of text on pages, presence of hyperlinks to other resources, etc. [1].

	1	2	3	4	5	
subitem not at all important	○	○	○	○	◉	essential

Clear selection

Does your paper address subitem 5-viii? *

Copy and paste relevant sections from the manuscript (include quotes in quotation marks "like this" to indicate direct quotes from your manuscript), or elaborate on this item by providing additional information not in the ms, or briefly explain why the item is not applicable/relevant for your study

Yes, each intervention arm was described. "Level 1 received educational materials on exercise and diet based on Center for Disease Control guidelines (24) that were delivered through a weekly email and linked to a resource directory on the study website (Multimedia Appendix 1). Level 2 received the same weekly educational email as Level 1, along with 3 pre-recorded videos every week of recommended exercise, accessible directly in the email or through the study website. Level 3 received the same resources provided to Level 1, along with three instructor-led group exercise classes per week that were attended with 5-8 Level 3 participants over cloud-based video conference (Zoom, San Jose, USA). " "The study website listed affiliation with the investigators' university, contained all resources referenced in the weekly email and was tailored to each participant's group assignment. Specifically, Level 1 could only view educational materials, Level 2 could view educational materials and exercise videos, while Level 3 could view educational materials and online class information (Multimedia Appendix 1)."

5-ix) Describe use parameters

Describe use parameters (e.g., intended "doses" and optimal timing for use). Clarify what instructions or recommendations were given to the user, e.g., regarding timing, frequency, heaviness of use, if any, or was the intervention used ad libitum.

	1	2	3	4	5	
subitem not at all important	○	○	○	◉	○	essential

Clear selection

Does your paper address subitem 5-ix?

Copy and paste relevant sections from the manuscript (include quotes in quotation marks "like this" to indicate direct quotes from your manuscript), or elaborate on this item by providing additional information not in the ms, or briefly explain why the item is not applicable/relevant for your study

Yes, participants were encouraged to exercise 3x/week using the device and mobile app. "All participants were provided a heart rate (HR) monitoring device and access to a mobile app (Myzone MZ-3, Chicago, IL) to track exercise intensity and were encouraged to exercise at least 3 sessions/week while wearing the device."

5-x) Clarify the level of human involvement

Clarify the level of human involvement (care providers or health professionals, also technical assistance) in the e-intervention or as co-intervention (detail number and expertise of professionals involved, if any, as well as “type of assistance offered, the timing and frequency of the support, how it is initiated, and the medium by which the assistance is delivered”. It may be necessary to distinguish between the level of human involvement required for the trial, and the level of human involvement required for a routine application outside of a RCT setting (discuss under item 21 – generalizability).

	1	2	3	4	5	
subitem not at all important	○	○	○	◉	○	essential

Clear selection

Does your paper address subitem 5-x?

Copy and paste relevant sections from the manuscript (include quotes in quotation marks "like this" to indicate direct quotes from your manuscript), or elaborate on this item by providing additional information not in the ms, or briefly explain why the item is not applicable/relevant for your study

Human involvement is described for each intervention arm, and also when technical support was needed. "Level 1 received educational materials on exercise and diet based on Center for Disease Control guidelines (24) that were delivered through a weekly email and linked to a resource directory on the study website (Multimedia Appendix 1). Level 2 received the same weekly educational email as Level 1, along with 3 pre-recorded videos every week of recommended exercise, accessible directly in the email or through the study website. Level 3 received the same resources provided to Level 1, along with three instructor-led group exercise classes per week that were attended with 5-8 Level 3 participants over cloud-based video conference (Zoom, San Jose, USA). " "Participants were also invited to email the study team if technical support was needed for any aspect of the intervention (HR monitor, app, study website, videos (Level 2) or videoconferencing (Level 3)). Technical support was carried out through email exchange or videoconference call, based on participant preference. "

5-xi) Report any prompts/reminders used

Report any prompts/reminders used: Clarify if there were prompts (letters, emails, phone calls, SMS) to use the application, what triggered them, frequency etc. It may be necessary to distinguish between the level of prompts/reminders required for the trial, and the level of prompts/reminders for a routine application outside of a RCT setting (discuss under item 21 – generalizability).

	1	2	3	4	5	
subitem not at all important	○	○	○	○	◉	essential

Clear selection

Does your paper address subitem 5-xi? *

Copy and paste relevant sections from the manuscript (include quotes in quotation marks "like this" to indicate direct quotes from your manuscript), or elaborate on this item by providing additional information not in the ms, or briefly explain why the item is not applicable/relevant for your study

All participants were given weekly email reminders, which is described under Intervention. "The MEPs summary was included in the weekly educational email to each participant through feedback on progress towards the recommended 300 MEPs/week, a reminder to perform PA and encouragement to interact with other participants on the Myzone app (Multimedia Appendix 1). "

5-xii) Describe any co-interventions (incl. training/support)

Describe any co-interventions (incl. training/support): Clearly state any interventions that are provided in addition to the targeted eHealth intervention, as ehealth intervention may not be designed as stand-alone intervention. This includes training sessions and support [1]. It may be necessary to distinguish between the level of training required for the trial, and the level of training for a routine application outside of a RCT setting (discuss under item 21 – generalizability.

	1	2	3	4	5	
subitem not at all important	○	○	○	○	◉	essential

Clear selection

Does your paper address subitem 5-xii? *

Copy and paste relevant sections from the manuscript (include quotes in quotation marks "like this" to indicate direct quotes from your manuscript), or elaborate on this item by providing additional information not in the ms, or briefly explain why the item is not applicable/relevant for your study

The 3 intervention arms are described under Intervention, along with technical support. "Level 1 received educational materials on exercise and diet based on Center for Disease Control guidelines (24) that were delivered through a weekly email and linked to a resource directory on the study website (Multimedia Appendix 1). Level 2 received the same weekly educational email as Level 1, along with 3 pre-recorded videos every week of recommended exercise, accessible directly in the email or through the study website. Level 3 received the same resources provided to Level 1, along with three instructor-led group exercise classes per week that were attended with 5-8 Level 3 participants over cloud-based video conference (Zoom, San Jose, USA). " "Participants were also invited to email the study team if technical support was needed for any aspect of the intervention (HR monitor, app, study website, videos (Level 2) or videoconferencing (Level 3)). Technical support was carried out through email exchange or videoconference call, based on participant preference. "

6a) Completely defined pre-specified primary and secondary outcome measures, including how and when they were assessed

Does your paper address CONSORT subitem 6a? *

Copy and paste relevant sections from the manuscript (include quotes in quotation marks "like this" to indicate direct quotes from your manuscript), or elaborate on this item by providing additional information not in the ms, or briefly explain why the item is not applicable/relevant for your study

Primary and secondary outcomes are described as follows: "The primary outcome of the study was feasibility, which was assessed through accrual, retention and adherence, and acceptability, which was assessed through qualitative evaluation. Accrual was defined as the time between enrollment of the first and last participant. Retention was defined as the percentage of participants completing post-intervention assessments. Adherence was defined as the observed to prescribed number of total exercise sessions completed using the MZ-3 (3 sessions/week x 8 weeks = 24 prescribed sessions). A priori, we considered the intervention feasible if the following criteria were satisfied (28): 1) accrual, ≥ 50% of eligible participants enrolled into the trial in a 1-year period, 2) retention, ≥ 70% of enrolled participants completing post-intervention assessments, 3) adherence, completion of ≥ 70% prescribed sessions (≥16 sessions). The acceptability of the intervention was assessed through feedback obtained at the end of the 8-week intervention through an investigator-developed web-based user experience survey. The survey asked participants to evaluate perceived ease of use and usefulness the mobile application, wearable sensor, emails, videos (Level 2), classes (Level 3), and overall satisfaction with the intervention through Likert scales (1= Strongly Agree, 5 = Strongly Disagree) and open-ended questions (Multimedia Appendix 3)." "Secondary Outcomes
The intervention was developed to support physical activity behavior changes that have been associated with improvements in cardiometabolic risk factors (26). Therefore, we explored changes in resting heart rate, self-reported physical activity level, quality of life and compliance as measured through exercise dose. All measurements were self-assessed remotely by participants at baseline and post-intervention except for exercise dose, which was monitored remotely and continuously throughout the intervention."

6a-i) Online questionnaires: describe if they were validated for online use and apply CHERRIES items to describe how the questionnaires were designed/deployed

If outcomes were obtained through online questionnaires, describe if they were validated for online use and apply CHERRIES items to describe how the questionnaires were designed/deployed [9].

	1	2	3	4	5	
subitem not at all important	○	○	○	◉	○	essential

Clear selection

Does your paper address subitem 6a-i?

Copy and paste relevant sections from manuscript text

Validated questionnaires were used to collect physical activity and quality of life data. Both questionnaires have been administered remotely in previous studies. "The IPAQ has been shown to have acceptable measurement properties, including repeatable data (pooled r=0.81 across data from 12 countries) and criterion validity comparable to other self-report methods (pooled r=0.3). The IPAQ was administered remotely using an online survey application (Qualtrics, Seattle, USA)." "QOL was assessed through the Short Form-36 (SF-36) (29) and administered remotely using an online survey application (Qualtrics). The SF-36 consists of four physical and four mental health components that quantify QOL in eight domains. " User experience data was collected from a survey developed by the study team. The CHERRIES items were applied to reporting of results. "The survey asked participants to evaluate perceived ease of use and usefulness the mobile application, wearable sensor, emails, videos (Level 2), classes (Level 3), and overall satisfaction with the intervention through Likert scales (1= Strongly Agree, 5 = Strongly Disagree) and open-ended questions (Multimedia Appendix 3)."

6a-ii) Describe whether and how “use” (including intensity of use/dosage) was defined/measured/monitored

Describe whether and how “use” (including intensity of use/dosage) was defined/measured/monitored (logins, logfile analysis, etc.). Use/adoption metrics are important process outcomes that should be reported in any ehealth trial.

	1	2	3	4	5	
subitem not at all important	○	○	○	○	◉	essential

Clear selection

Does your paper address subitem 6a-ii?

Copy and paste relevant sections from manuscript text

We used adherence and compliance to the exercise intervention to quantify intervention dose, and retention to quantify adoption. "Retention was defined as the percentage of participants completing post-intervention assessments. Adherence was defined as the observed to prescribed number of total exercise sessions completed using the MZ-3 (3 sessions/week x 8 weeks = 24 prescribed sessions). A priori, we considered the intervention feasible if the following criteria were satisfied (28): 1) accrual, ≥ 50% of eligible participants enrolled into the trial in a 1-year period, 2) retention, ≥ 70% of enrolled participants completing post-intervention assessments, 3) adherence, completion of ≥ 70% prescribed sessions (≥16 sessions). "

6a-iii) Describe whether, how, and when qualitative feedback from participants was obtained

Describe whether, how, and when qualitative feedback from participants was obtained (e.g., through emails, feedback forms, interviews, focus groups).

	1	2	3	4	5	
subitem not at all important	○	○	○	○	◉	essential

Clear selection

Does your paper address subitem 6a-iii?

Copy and paste relevant sections from manuscript text

We collected qualitative feedback to assess acceptability. "The acceptability of the intervention was assessed through feedback obtained at the end of the 8-week intervention through an investigator-developed web-based user experience survey. The survey asked participants to evaluate perceived ease of use and usefulness the mobile application, wearable sensor, emails, videos (Level 2), classes (Level 3), and overall satisfaction with the intervention through Likert scales (1= Strongly Agree, 5 = Strongly Disagree) and open-ended questions (Multimedia Appendix 3)."

6b) Any changes to trial outcomes after the trial commenced, with reasons

Does your paper address CONSORT subitem 6b? *

Copy and paste relevant sections from the manuscript (include quotes in quotation marks "like this" to indicate direct quotes from your manuscript), or elaborate on this item by providing additional information not in the ms, or briefly explain why the item is not applicable/relevant for your study

Outcomes were not changed after the trial commenced.

7a) How sample size was determined

NPT: When applicable, details of whether and how the clustering by care provides or centers was addressed

7a-i) Describe whether and how expected attrition was taken into account when calculating the sample size

Describe whether and how expected attrition was taken into account when calculating the sample size.

	1	2	3	4	5	
subitem not at all important	○	○	○	○	◉	essential

Clear selection

Does your paper address subitem 7a-i?

Copy and paste relevant sections from manuscript title (include quotes in quotation marks "like this" to indicate direct quotes from your manuscript), or elaborate on this item by providing additional information not in the ms, or briefly explain why the item is not applicable/relevant for your study

Sample size and attrition are described under the section on Power. "The sample size for a two-group feasibility pilot trial is recommended to be at least n=50 (25 participants per group) (30). Our estimated sample size for this pilot study was 75 participants (25 participants per group). Accounting for 10% attrition, we recruited a sample size of n=84. "

7b) When applicable, explanation of any interim analyses and stopping guidelines

Does your paper address CONSORT subitem 7b? *

Copy and paste relevant sections from the manuscript (include quotes in quotation marks "like this" to indicate direct quotes from your manuscript), or elaborate on this item by providing additional information not in the ms, or briefly explain why the item is not applicable/relevant for your study

An interim analysis nor stopping guidelines were not planned.

8a) Method used to generate the random allocation sequence

NPT: When applicable, how care providers were allocated to each trial group

Does your paper address CONSORT subitem 8a? *

Copy and paste relevant sections from the manuscript (include quotes in quotation marks "like this" to indicate direct quotes from your manuscript), or elaborate on this item by providing additional information not in the ms, or briefly explain why the item is not applicable/relevant for your study

Computer-generated block randomization was used. "Participants were allocated (1:1:1) to increasing levels of behavioral support (Levels 1-3) using computer-generated block randomization with blocks that randomly varied in sizes of 3, 6, 9 or 12. "

8b) Type of randomisation; details of any restriction (such as blocking and block size)

Does your paper address CONSORT subitem 8b? *

Copy and paste relevant sections from the manuscript (include quotes in quotation marks "like this" to indicate direct quotes from your manuscript), or elaborate on this item by providing additional information not in the ms, or briefly explain why the item is not applicable/relevant for your study

Computer-generated block randomization was used. "Participants were allocated (1:1:1) to increasing levels of behavioral support (Levels 1-3) using computer-generated block randomization with blocks that randomly varied in sizes of 3, 6, 9 or 12. "

9) Mechanism used to implement the random allocation sequence (such as sequentially numbered containers), describing any steps taken to conceal the sequence until interventions were assigned

Does your paper address CONSORT subitem 9? *

Copy and paste relevant sections from the manuscript (include quotes in quotation marks "like this" to indicate direct quotes from your manuscript), or elaborate on this item by providing additional information not in the ms, or briefly explain why the item is not applicable/relevant for your study

The randomization list was prepared and implemented by an investigator who was uninvolved in all other study procedures. "The randomization list was prepared in advance by an investigator (HLT) uninvolved with any other study procedures. "

10) Who generated the random allocation sequence, who enrolled participants, and who assigned participants to interventions

Does your paper address CONSORT subitem 10? *

Copy and paste relevant sections from the manuscript (include quotes in quotation marks "like this" to indicate direct quotes from your manuscript), or elaborate on this item by providing additional information not in the ms, or briefly explain why the item is not applicable/relevant for your study

The randomization list was prepared and implemented by an investigator who was uninvolved in all other study procedures. "The randomization list was prepared in advance by an investigator (HLT) uninvolved with any other study procedures. After consent and enrollment procedures, the allocation assignment was requested from HLT. "

11a) If done, who was blinded after assignment to interventions (for example, participants, care providers, those assessing outcomes) and how

NPT: Whether or not administering co-interventions were blinded to group assignment

11a-i) Specify who was blinded, and who wasn’t

Specify who was blinded, and who wasn’t. Usually, in web-based trials it is not possible to blind the participants [1, 3] (this should be clearly acknowledged), but it may be possible to blind outcome assessors, those doing data analysis or those administering co-interventions (if any).

	1	2	3	4	5	
subitem not at all important	○	○	○	◉	○	essential

Clear selection

Does your paper address subitem 11a-i? *

Copy and paste relevant sections from the manuscript (include quotes in quotation marks "like this" to indicate direct quotes from your manuscript), or elaborate on this item by providing additional information not in the ms, or briefly explain why the item is not applicable/relevant for your study

Investigators involved in study procedures were not blinded. Participants were not blinded to their intevention but were unaware of what was received relative to other study arms and did not interact with participants from other study arms. "Participants were told that they would be randomly assigned to receive certain resources but were unaware of what was received relative to other study arms. "

11a-ii) Discuss e.g., whether participants knew which intervention was the “intervention of interest” and which one was the “comparator”

Informed consent procedures (4a-ii) can create biases and certain expectations - discuss e.g., whether participants knew which intervention was the “intervention of interest” and which one was the “comparator”.

	1	2	3	4	5	
subitem not at all important	○	○	○	○	◉	essential

Clear selection

Does your paper address subitem 11a-ii?

Copy and paste relevant sections from the manuscript (include quotes in quotation marks "like this" to indicate direct quotes from your manuscript), or elaborate on this item by providing additional information not in the ms, or briefly explain why the item is not applicable/relevant for your study

Participants were aware of the 3 intervention arms as this was described in the consent. However, participants were unaware of what was received relative to other study arms. "Participants were told that they would be randomly assigned to receive certain resources but were unaware of what was received relative to other study arms. "

11b) If relevant, description of the similarity of interventions

(this item is usually not relevant for ehealth trials as it refers to similarity of a placebo or sham intervention to a active medication/intervention)

Does your paper address CONSORT subitem 11b? *

Copy and paste relevant sections from the manuscript (include quotes in quotation marks "like this" to indicate direct quotes from your manuscript), or elaborate on this item by providing additional information not in the ms, or briefly explain why the item is not applicable/relevant for your study

All participants received the Level 1 intervention, while Level 2 and Level 3 intervention arms were additive on Level 1. "Level 1 received educational materials on exercise and diet based on Center for Disease Control guidelines (24) that were delivered through a weekly email and linked to a resource directory on the study website (Multimedia Appendix 1). Level 2 received the same weekly educational email as Level 1, along with 3 pre-recorded videos every week of recommended exercise, accessible directly in the email or through the study website. Level 3 received the same resources provided to Level 1, along with three instructor-led group exercise classes per week that were attended with 5-8 Level 3 participants over cloud-based video conference (Zoom, San Jose, USA). All participants were provided a heart rate (HR) monitoring device and access to a mobile app (Myzone MZ-3, Chicago, IL) to track exercise intensity and were encouraged to exercise at least 3 sessions/week while wearing the device. "

12a) Statistical methods used to compare groups for primary and secondary outcomes

NPT: When applicable, details of whether and how the clustering by care providers or centers was addressed

Does your paper address CONSORT subitem 12a? *

Copy and paste relevant sections from the manuscript (include quotes in quotation marks "like this" to indicate direct quotes from your manuscript), or elaborate on this item by providing additional information not in the ms, or briefly explain why the item is not applicable/relevant for your study

This is described under Statistical Analysis. "Descriptive statistics were used to summarize participant characteristics, feasibility, acceptability and secondary outcomes. Proportions were calculated for categorical variables and median (IQR) for continuous variables. Comparisons of baseline characteristics and differences between groups were made using Kruskal-Wallis rank sum tests for continuous outcomes and Dunn tests with Bonferroni adjustment for post hoc testing. "

12a-i) Imputation techniques to deal with attrition / missing values

Imputation techniques to deal with attrition / missing values: Not all participants will use the intervention/comparator as intended and attrition is typically high in ehealth trials. Specify how participants who did not use the application or dropped out from the trial were treated in the statistical analysis (a complete case analysis is strongly discouraged, and simple imputation techniques such as LOCF may also be problematic [4]).

	1	2	3	4	5	
subitem not at all important	○	○	○	○	◉	essential

Clear selection

Does your paper address subitem 12a-i? *

Copy and paste relevant sections from the manuscript (include quotes in quotation marks "like this" to indicate direct quotes from your manuscript), or elaborate on this item by providing additional information not in the ms, or briefly explain why the item is not applicable/relevant for your study

Described under Statistical Analysis. "For within-group baseline to post-intervention comparisons, intent-to-treat models were used to compute the difference (post-pre) on individual scores and Wilcoxon signed rank tests with Bonferroni correction were used to assess significance."

12b) Methods for additional analyses, such as subgroup analyses and adjusted analyses

Does your paper address CONSORT subitem 12b? *

Copy and paste relevant sections from the manuscript (include quotes in quotation marks "like this" to indicate direct quotes from your manuscript), or elaborate on this item by providing additional information not in the ms, or briefly explain why the item is not applicable/relevant for your study

Subgroup or adjusted analyses were not performed.

X26) REB/IRB Approval and Ethical Considerations [recommended as subheading under "Methods"] (not a CONSORT item)

X26-i) Comment on ethics committee approval

	1	2	3	4	5	
subitem not at all important	○	○	○	○	◉	essential

Clear selection

Does your paper address subitem X26-i?

Copy and paste relevant sections from the manuscript (include quotes in quotation marks "like this" to indicate direct quotes from your manuscript), or elaborate on this item by providing additional information not in the ms, or briefly explain why the item is not applicable/relevant for your study

IRB approval was obtained prior to and throughout the trial. "Ethics approval [1542178-5] was obtained from the California State University, Long Beach Institutional Review Board for the Protection of Human Subjects prior to study commencement and maintained throughout the trial."

x26-ii) Outline informed consent procedures

Outline informed consent procedures e.g., if consent was obtained offline or online (how? Checkbox, etc.?), and what information was provided (see 4a-ii). See [6] for some items to be included in informed consent documents.

	1	2	3	4	5	
subitem not at all important	○	○	○	○	◉	essential

Clear selection

Does your paper address subitem X26-ii?

Copy and paste relevant sections from the manuscript (include quotes in quotation marks "like this" to indicate direct quotes from your manuscript), or elaborate on this item by providing additional information not in the ms, or briefly explain why the item is not applicable/relevant for your study

Described under "Randomization and Blinding". "All participants provided online written consent prior to randomization."

X26-iii) Safety and security procedures

Safety and security procedures, incl. privacy considerations, and any steps taken to reduce the likelihood or detection of harm (e.g., education and training, availability of a hotline)

	1	2	3	4	5	
subitem not at all important	○	○	○	○	○	essential

Does your paper address subitem X26-iii?

Copy and paste relevant sections from the manuscript (include quotes in quotation marks "like this" to indicate direct quotes from your manuscript), or elaborate on this item by providing additional information not in the ms, or briefly explain why the item is not applicable/relevant for your study

The informed consent described steps to take if a participant was injured while exercising. Privacy was addressed under Intervention. "Participants were invited to use a psuedoname on the mobile app, rather than their real name or email, if they preferred. "

RESULTS

13a) For each group, the numbers of participants who were randomly assigned, received intended treatment, and were analysed for the primary outcome

NPT: The number of care providers or centers performing the intervention in each group and the number of patients treated by each care provider in each center

Does your paper address CONSORT subitem 13a? *

Copy and paste relevant sections from the manuscript (include quotes in quotation marks "like this" to indicate direct quotes from your manuscript), or elaborate on this item by providing additional information not in the ms, or briefly explain why the item is not applicable/relevant for your study

CONSORT information is provided in Figure 1 via a flow diagram.

13b) For each group, losses and exclusions after randomisation, together with reasons

Does your paper address CONSORT subitem 13b? (NOTE: Preferably, this is shown in a CONSORT flow diagram) *

Copy and paste relevant sections from the manuscript (include quotes in quotation marks "like this" to indicate direct quotes from your manuscript), or elaborate on this item by providing additional information not in the ms, or briefly explain why the item is not applicable/relevant for your study

CONSORT information is provided in Figure 1 via a flow diagram. Reasons for withdrawal are also described under Participants in the results. "For the 14 participants that withdrew after randomization, time commitment or schedule conflict were the primary reasons stated for withdrawal. Withdrawal was similar across groups with n=5 withdrawals in level 1, n=3 withdrawals in level 2 and n=4 withdrawals in level 3."

13b-i) Attrition diagram

Strongly recommended: An attrition diagram (e.g., proportion of participants still logging in or using the intervention/comparator in each group plotted over time, similar to a survival curve) or other figures or tables demonstrating usage/dose/engagement.

	1	2	3	4	5	
subitem not at all important	○	○	○	○	◉	essential

Clear selection

Does your paper address subitem 13b-i?

Copy and paste relevant sections from the manuscript or cite the figure number if applicable (include quotes in quotation marks "like this" to indicate direct quotes from your manuscript), or elaborate on this item by providing additional information not in the ms, or briefly explain why the item is not applicable/relevant for your study

Adherence and compliance between groups are graphed in Figure 3.

14a) Dates defining the periods of recruitment and follow-up

Does your paper address CONSORT subitem 14a? *

Copy and paste relevant sections from the manuscript (include quotes in quotation marks "like this" to indicate direct quotes from your manuscript), or elaborate on this item by providing additional information not in the ms, or briefly explain why the item is not applicable/relevant for your study

Recruitment period and followup (via retention) is defined under Participants in the results. "A total of 204 potential participants expressed interest in the study via a web form between August 2020 and August 2021 (Figure 1). " "A total of 70 participants completed the 8-week intervention (n=19 in level 1, n=27 in level 2, and n=24 in level 3). "

14a-i) Indicate if critical “secular events” fell into the study period

Indicate if critical “secular events” fell into the study period, e.g., significant changes in Internet resources available or “changes in computer hardware or Internet delivery resources”

	1	2	3	4	5	
subitem not at all important	(•)	( )	( )	( )	( )	essential

Clear selection

Does your paper address subitem 14a-i?

Copy and paste relevant sections from the manuscript (include quotes in quotation marks "like this" to indicate direct quotes from your manuscript), or elaborate on this item by providing additional information not in the ms, or briefly explain why the item is not applicable/relevant for your study

No secular events occurred during the study or were noted in the manuscript.

14b) Why the trial ended or was stopped (early)

Does your paper address CONSORT subitem 14b? *

Copy and paste relevant sections from the manuscript (include quotes in quotation marks "like this" to indicate direct quotes from your manuscript), or elaborate on this item by providing additional information not in the ms, or briefly explain why the item is not applicable/relevant for your study

As this was a pilot trial, we aimed to evaluate feasibility on at least n=75 participants. We recruited over this amount to account for attrition (n=84) and stopped the trial after this sample size was reached.

15) A table showing baseline demographic and clinical characteristics for each group

NPT: When applicable, a description of care providers (case volume, qualification, expertise, etc.) and centers (volume) in each group

Does your paper address CONSORT subitem 15? *

Copy and paste relevant sections from the manuscript (include quotes in quotation marks "like this" to indicate direct quotes from your manuscript), or elaborate on this item by providing additional information not in the ms, or briefly explain why the item is not applicable/relevant for your study

Baseline characteristics of participants are provided in Table 3.

15-i) Report demographics associated with digital divide issues

In ehealth trials it is particularly important to report demographics associated with digital divide issues, such as age, education, gender, social-economic status, computer/Internet/ehealth literacy of the participants, if known.

	1	2	3	4	5	
subitem not at all important	○	○	○	◉	○	essential

Clear selection

Does your paper address subitem 15-i? *

Copy and paste relevant sections from the manuscript (include quotes in quotation marks "like this" to indicate direct quotes from your manuscript), or elaborate on this item by providing additional information not in the ms, or briefly explain why the item is not applicable/relevant for your study

All demographics listed above except digital literacy are reported in Table 3.

16) For each group, number of participants (denominator) included in each analysis and whether the analysis was by original assigned groups

16-i) Report multiple “denominators” and provide definitions

Report multiple “denominators” and provide definitions: Report N’s (and effect sizes) “across a range of study participation [and use] thresholds” [1], e.g., N exposed, N consented, N used more than x times, N used more than y weeks, N participants “used” the intervention/comparator at specific pre-defined time points of interest (in absolute and relative numbers per group). Always clearly define “use” of the intervention.

	1	2	3	4	5	
subitem not at all important	○	○	○	○	◉	essential

Clear selection

Does your paper address subitem 16-i? *

Copy and paste relevant sections from the manuscript (include quotes in quotation marks "like this" to indicate direct quotes from your manuscript), or elaborate on this item by providing additional information not in the ms, or briefly explain why the item is not applicable/relevant for your study

This information is provided in the CONSORT flow (Figure 1) and described under feasibility outcomes. Effect sizes are not provided given the nonparametric analyses. "We found accrual to be feasible as 62.2% (84/135) of eligible participants enrolled in the study within a 1-year period. For retention, 50% (13/26) of Level 1, 60% (18/30) of Level 2 and 82.1% (23/28) of Level 3 participants completed the post-intervention assessments. As our threshold for retention was ≥ 70% of enrolled participants completing post-intervention assessments, only the level 3 intervention met retention criteria. "

16-ii) Primary analysis should be intent-to-treat

Primary analysis should be intent-to-treat, secondary analyses could include comparing only “users”, with the appropriate caveats that this is no longer a randomized sample (see 18-i).

	1	2	3	4	5	
subitem not at all important	○	○	○	○	●	essential

Clear selection

Does your paper address subitem 16-ii?

Copy and paste relevant sections from the manuscript (include quotes in quotation marks "like this" to indicate direct quotes from your manuscript), or elaborate on this item by providing additional information not in the ms, or briefly explain why the item is not applicable/relevant for your study

Intent-to-treat was the primary analysis for outcomes with pre- to post- data. "For within-group baseline to post-intervention comparisons, intent-to-treat models were used to compute the difference (post-pre) on individual scores and Wilcoxon signed rank tests with Bonferroni correction were used to assess significance"

17a) For each primary and secondary outcome, results for each group, and the estimated effect size and its precision (such as 95% confidence interval)

Does your paper address CONSORT subitem 17a? *

Copy and paste relevant sections from the manuscript (include quotes in quotation marks "like this" to indicate direct quotes from your manuscript), or elaborate on this item by providing additional information not in the ms, or briefly explain why the item is not applicable/relevant for your study

Results are provided for each group. However, nonparametric analyses were undertaken due to the non-normality of the data. Therefore, data are reported as median [IQR] and effect sizes are not calculated. "Between-group comparisons in the change from baseline to week 8 for self-reported PA, resting heart rate and quality of life are shown in Figure 2, while median (IQR) values and within-group changes are presented in Table 5. For self-reported PA, weekly MVPA time increased from baseline to 8 weeks in Level 2 ($p=.003$) and Level 3 ($p<.001$), but not in Level 1 (Table 3). "

17a-i) Presentation of process outcomes such as metrics of use and intensity of use

In addition to primary/secondary (clinical) outcomes, the presentation of process outcomes such as metrics of use and intensity of use (dose, exposure) and their operational definitions is critical. This does not only refer to metrics of attrition (13-b) (often a binary variable), but also to more continuous exposure metrics such as “average session length”. These must be accompanied by a technical description how a metric like a “session” is defined (e.g., timeout after idle time) [1] (report under item 6a).

	1	2	3	4	5	
subitem not at all important	○	○	○	○	○	essential

Does your paper address subitem 17a-i?

Copy and paste relevant sections from the manuscript (include quotes in quotation marks "like this" to indicate direct quotes from your manuscript), or elaborate on this item by providing additional information not in the ms, or briefly explain why the item is not applicable/relevant for your study

Attrition,adherence and compliance (time x intensity) to the intervention (Exercise) is reported. "Between-group comparisons for total and weekly exercise dose are shown in Figure 3. Significant differences were observed between Level 3 and Level 1 for total exercise dose (Level 1: 257 [0-1258] MEPs vs. Level 3: 2018 [1167-2810] MEPs; p=.002), weekly exercise dose (Level 1: 32 [0-157] MEPS/wk vs. Level 3: 252 [146-351] MEPS/wk; p=.003), total time spent in moderate-vigorous exercise (Level 1: 81 [0-600] min vs. Level 3: 767 [355-1072] min; p=.002), weekly time spent in moderate-vigorous exercise (Level 1: 9 [0-67] min/wk vs. Level 3: 85 [39-119] min/wk; p=.002), total exercise sessions (Level 1: 5 [0-17] sessions vs. Level 3: 24 [13-28] sessions; p=.01) and weekly exercise sessions (Level 1: 0.5 [0-2] sessions/wk vs. Level 3: 2.7 [1-3] sessions/wk; p=.01). ."

17b) For binary outcomes, presentation of both absolute and relative effect sizes is recommended

Does your paper address CONSORT subitem 17b? *

Copy and paste relevant sections from the manuscript (include quotes in quotation marks "like this" to indicate direct quotes from your manuscript), or elaborate on this item by providing additional information not in the ms, or briefly explain why the item is not applicable/relevant for your study

Nonparametric analyses were undertaken due to the non-normality of the data. Therefore, data are reported as median [IQR] and effect sizes are not calculated.

18) Results of any other analyses performed, including subgroup analyses and adjusted analyses, distinguishing pre-specified from exploratory

Does your paper address CONSORT subitem 18? *

Copy and paste relevant sections from the manuscript (include quotes in quotation marks "like this" to indicate direct quotes from your manuscript), or elaborate on this item by providing additional information not in the ms, or briefly explain why the item is not applicable/relevant for your study

Subgroup and adjusted analyses were not performed.

18-i) Subgroup analysis of comparing only users

A subgroup analysis of comparing only users is not uncommon in ehealth trials, but if done, it must be stressed that this is a self-selected sample and no longer an unbiased sample from a randomized trial (see 16-iii).

	1	2	3	4	5	
subitem not at all important	○	○	◉	○	○	essential

Clear selection

Does your paper address subitem 18-i?

Copy and paste relevant sections from the manuscript (include quotes in quotation marks "like this" to indicate direct quotes from your manuscript), or elaborate on this item by providing additional information not in the ms, or briefly explain why the item is not applicable/relevant for your study

Subgroup and adjusted analyses were not performed.

19) All important harms or unintended effects in each group

(for specific guidance see CONSORT for harms)

Does your paper address CONSORT subitem 19? *

Copy and paste relevant sections from the manuscript (include quotes in quotation marks "like this" to indicate direct quotes from your manuscript), or elaborate on this item by providing additional information not in the ms, or briefly explain why the item is not applicable/relevant for your study

No harms or adverse events were reported. "No adverse events or privacy breaches were reported throughout the trial period."

19-i) Include privacy breaches, technical problems

Include privacy breaches, technical problems. This does not only include physical “harm” to participants, but also incidents such as perceived or real privacy breaches [1], technical problems, and other unexpected/unintended incidents. “Unintended effects” also includes unintended positive effects [2].

	1	2	3	4	5	
subitem not at all important	○	○	◉	○	○	essential

Clear selection

Does your paper address subitem 19-i?

Copy and paste relevant sections from the manuscript (include quotes in quotation marks "like this" to indicate direct quotes from your manuscript), or elaborate on this item by providing additional information not in the ms, or briefly explain why the item is not applicable/relevant for your study

No privacy breaches were reported. "No adverse events or privacy breaches were reported throughout the trial period."

19-ii) Include qualitative feedback from participants or observations from staff/researchers

Include qualitative feedback from participants or observations from staff/researchers, if available, on strengths and shortcomings of the application, especially if they point to unintended/unexpected effects or uses. This includes (if available) reasons for why people did or did not use the application as intended by the developers.

	1	2	3	4	5	
subitem not at all important	○	○	○	○	◉	essential

Clear selection

Does your paper address subitem 19-ii?

Copy and paste relevant sections from the manuscript (include quotes in quotation marks "like this" to indicate direct quotes from your manuscript), or elaborate on this item by providing additional information not in the ms, or briefly explain why the item is not applicable/relevant for your study

Qualitative feedback was summarized under "Acceptability" (Table 4 and in the narrative). "Of the 37 participants who completed the user experience survey, 30 (n=6 Level 1, n=10 Level 2, n=14 Level 3) ..."

DISCUSSION

22) Interpretation consistent with results, balancing benefits and harms, and considering other relevant evidence

NPT: In addition, take into account the choice of the comparator, lack of or partial blinding, and unequal expertise of care providers or centers in each group

22-i) Restate study questions and summarize the answers suggested by the data, starting with primary outcomes and process outcomes (use)

Restate study questions and summarize the answers suggested by the data, starting with primary outcomes and process outcomes (use).

	1	2	3	4	5	
subitem not at all important	○	○	○	○	●	essential

Clear selection

Does your paper address subitem 22-i? *

Copy and paste relevant sections from the manuscript (include quotes in quotation marks "like this" to indicate direct quotes from your manuscript), or elaborate on this item by providing additional information not in the ms, or briefly explain why the item is not applicable/relevant for your study

Described under Principal Results
"The primary aim of this pilot, randomized controlled trial was to evaluate the feasibility and acceptability of an mHealth intervention with additive levels of behavioral support. The secondary aim explored differences in resting heart rate, self-reported PA, quality of life and exercise dose between the intervention levels. "

22-ii) Highlight unanswered new questions, suggest future research

Highlight unanswered new questions, suggest future research.

	1	2	3	4	5	
subitem not at all important	○	○	○	○	●	essential

Clear selection

Does your paper address subitem 22-ii?

Copy and paste relevant sections from the manuscript (include quotes in quotation marks "like this" to indicate direct quotes from your manuscript), or elaborate on this item by providing additional information not in the ms, or briefly explain why the item is not applicable/relevant for your study

Described at the end of each Discussion paragraph and summarized in the Conclusion. "Future, appropriately powered trials should investigate the effectiveness of videoconference interventions on health-related outcomes. More research is needed to elucidate feasible exercise interventions using asynchronous components, especially in diverse populations."

20) Trial limitations, addressing sources of potential bias, imprecision, and, if relevant, multiplicity of analyses

20-i) Typical limitations in ehealth trials

Typical limitations in ehealth trials: Participants in ehealth trials are rarely blinded. Ehealth trials often look at a multiplicity of outcomes, increasing risk for a Type I error. Discuss biases due to non-use of the intervention/usability issues, biases through informed consent procedures, unexpected events.

	1	2	3	4	5	
subitem not at all important	○	○	○	○	●	essential

Clear selection

Does your paper address subitem 20-i? *

Copy and paste relevant sections from the manuscript (include quotes in quotation marks "like this" to indicate direct quotes from your manuscript), or elaborate on this item by providing additional information not in the ms, or briefly explain why the item is not applicable/relevant for your study

Addressed in Limitations. "There are several limitations to this study that may have influenced findings. First, individuals who volunteered for the study were primarily young-to-middle aged adults with access to mobile devices and high-speed internet. Thus, the results may not be generalizable to all adults and those with low technology literacy or access. A second limitation is that we did not include a second method of objectively measured PA besides the HR monitor. Because PA adherence was only recorded when the HR monitor was worn, assessment of PA through a continuously worn accelerometer could have provided additional insight into non-exercise physical activity. Thus, the PA recorded in this study was likely an underestimate of overall activity. A third limitation is the lack of assessment of health-related outcomes. While the primary aim of this study was feasibility, measurement of clinical outcomes could provide effect sizes for a fully powered RCT. Last, a follow-up assessment after the intervention would have been helpful to evaluate the sustainability of the PA adherence beyond the 8-week intervention. "

21) Generalisability (external validity, applicability) of the trial findings

NPT: External validity of the trial findings according to the intervention, comparators, patients, and care providers or centers involved in the trial

21-i) Generalizability to other populations

Generalizability to other populations: In particular, discuss generalizability to a general Internet population, outside of a RCT setting, and general patient population, including applicability of the study results for other organizations

	1	2	3	4	5	
subitem not at all important	○	○	○	○	○	essential

Does your paper address subitem 21-i?

Copy and paste relevant sections from the manuscript (include quotes in quotation marks "like this" to indicate direct quotes from your manuscript), or elaborate on this item by providing additional information not in the ms, or briefly explain why the item is not applicable/relevant for your study

Described under Conclusions. "In summary, our randomized controlled pilot trial demonstrates that a videoconference mHealth intervention is feasible and can promote significant increases in PA in apparently healthy, inactive adults."

21-ii) Discuss if there were elements in the RCT that would be different in a routine application setting

Discuss if there were elements in the RCT that would be different in a routine application setting (e.g., prompts/reminders, more human involvement, training sessions or other co-interventions) and what impact the omission of these elements could have on use, adoption, or outcomes if the intervention is applied outside of a RCT setting.

	1	2	3	4	5	
subitem not at all important	○	○	○	○	●	essential

Clear selection

Does your paper address subitem 21-ii?

Copy and paste relevant sections from the manuscript (include quotes in quotation marks "like this" to indicate direct quotes from your manuscript), or elaborate on this item by providing additional information not in the ms, or briefly explain why the item is not applicable/relevant for your study

Described throughout the Discussion. "Given that the unsupervised, asynchronous format is a popular format used in healthcare settings for preventative and outpatient rehabilitative programs, future studies that incorporate asynchronous delivery should first employ formative approaches, such as usability testing or community-based participatory research, to inform the development of the intervention with user feedback. "

OTHER INFORMATION

23) Registration number and name of trial registry

Does your paper address CONSORT subitem 23? *

Copy and paste relevant sections from the manuscript (include quotes in quotation marks "like this" to indicate direct quotes from your manuscript), or elaborate on this item by providing additional information not in the ms, or briefly explain why the item is not applicable/relevant for your study

Reported in abstract and in Methods. "We conducted a 3-arm randomized pilot trial of an 8-week intervention for physically inactive adults (ClinicalTrials.gov NCT05192421) "

24) Where the full trial protocol can be accessed, if available

Does your paper address CONSORT subitem 24? *

Cite a Multimedia Appendix, other reference, or copy and paste relevant sections from the manuscript (include quotes in quotation marks "like this" to indicate direct quotes from your manuscript), or elaborate on this item by providing additional information not in the ms, or briefly explain why the item is not applicable/relevant for your study

A trial protocol was not published for this pilot study.

25) Sources of funding and other support (such as supply of drugs), role of funders

Does your paper address CONSORT subitem 25? *

Copy and paste relevant sections from the manuscript (include quotes in quotation marks "like this" to indicate direct quotes from your manuscript), or elaborate on this item by providing additional information not in the ms, or briefly explain why the item is not applicable/relevant for your study

Under Acknowledgements, "We thank the Myzone company for providing the heart rate monitors and cloud-based interface for the intervention, and the CSULB College of Health and Human Services for financial support."

X27) Conflicts of Interest (not a CONSORT item)

X27-i) State the relation of the study team towards the system being evaluated

In addition to the usual declaration of interests (financial or otherwise), also state the relation of the study team towards the system being evaluated, i.e., state if the authors/evaluators are distinct from or identical with the developers/sponsors of the intervention.

	1	2	3	4	5	
subitem not at all important	○	○	○	◉	○	essential

Clear selection

Does your paper address subitem X27-i?

Copy and paste relevant sections from the manuscript (include quotes in quotation marks "like this" to indicate direct quotes from your manuscript), or elaborate on this item by providing additional information not in the ms, or briefly explain why the item is not applicable/relevant for your study

No conflicts of interest are reported.

About the CONSORT EHEALTH checklist

As a result of using this checklist, did you make changes in your manuscript? *

- ○ yes, major changes
- ◉ yes, minor changes
- ○ no

What were the most important changes you made as a result of using this checklist?

Not any one important change, but it was useful to check to make sure these items were clearly stated.

How much time did you spend on going through the checklist INCLUDING making changes in your manuscript *

A lot of time - 8 hours. Also, answers on the form ended up being too long, so I had to go back and reformat.

As a result of using this checklist, do you think your manuscript has improved? *

- ◉ yes
- ○ no
- ○ Other:

Would you like to become involved in the CONSORT EHEALTH group?

This would involve for example becoming involved in participating in a workshop and writing an "Explanation and Elaboration" document

- ○ yes
- ○ no
- ◉ Other: maybe. Would like to know more about it.

Clear selection

Any other comments or questions on CONSORT EHEALTH

Your answer

STOP - Save this form as PDF before you click submit

To generate a record that you filled in this form, we recommend to generate a PDF of this page (on a Mac, simply select "print" and then select "print as PDF") before you submit it.

When you submit your (revised) paper to JMIR, please upload the PDF as supplementary file.

Don't worry if some text in the textboxes is cut off, as we still have the complete information in our database. Thank you!

Final step: Click submit !

Click submit so we have your answers in our database!

Submit Clear form

Never submit passwords through Google Forms.

This content is neither created nor endorsed by Google. Report Abuse - Terms of Service - Privacy Policy

Google Forms
